# Ensiled Wet Storage Accelerates Pretreatment for Bioconversion of Corn Stover

**DOI:** 10.3389/fbioe.2018.00195

**Published:** 2018-12-14

**Authors:** Dzidzor Essien, Tom L. Richard

**Affiliations:** Department of Agricultural and Biological Engineering, Pennsylvania State University, University Park, PA, United States

**Keywords:** acetic acid, biofuel, biomass, fermentation, inhibitors, pretreatment, wet storage, ensilage

## Abstract

Organic acids produced during ensiled wet storage are beneficial during the storage process, both for biomass preservation, and to aid in mild *in-situ* pretreatment. However, there is concern these acids could later have negative impacts on downstream processes, especially microbial fermentation. Organic acids can inhibit microbial metabolism or growth, which in turn could affect biofuel productivity or yield. This study investigated the interaction of organic acids produced during ensiled storage with subsequent pretreatment of the resulting corn stover silage, as well as the potential for interference with downstream ethanol fermentation. Interaction with pretreatment was observed by measuring xylan and glucan removal and the formation of inhibitors. The results indicated that organic acids generally do not impede downstream processes and in fact can be beneficial. The levels of organic acids produced during 220 days of storage jar tests at 23°C or 37°C, and their transformation during pretreatment, remained below inhibitory levels. Concentrations of individual acids did not exceed 6 g per liter of the pretreated volume, and < 5% on a dry matter basis. Whereas, unensiled corn stover required 15 min of 190°C pretreatment to optimize sugar release, ensiled corn stover could be treated equally effectively at a lower pretreatment duration of 10 min. Furthermore, the different organic acid profiles that accumulate at various storage moisture levels (35–65%) do not differ significantly in their impact on downstream ethanol fermentation. These results indicate biorefineries using ensiled corn stover feedstock at 35–65% moisture levels can expect as good or better biofuel yields as with unensiled stover, while reducing pretreatment costs.

## Introduction

Wet storage is the storage of biomass materials under anaerobic conditions that inhibit microbial biodegradation. This is most frequently accomplished by creating an oxygen barrier (a silo, tarp, etc.) and storing the biomass at moisture levels that permit acidogenic anaerobic microorganisms to grow and produce sufficient quantities of organic acids to reduce pH to levels below pH 5, where very little degradation occurs. This microbially enhanced wet storage process, also known as ensilage, has long been used for storage of herbaceous plants for livestock feeds, allowing long term biomass preservation. There are other wet storage systems that do not rely on *in-situ* microbial organic acid production, instead adding external acids, or alkali compounds to adjust pH or use other biocide strategies to reduce degradation. The primary alternative to wet storage is dry storage, which requires keeping moisture levels low enough—usually below 20%—to slow down and inhibit active microbial activity. Traditional dry storage of biomass feedstocks in bales and other formats is low cost and can be effective if the materials are kept dry but carries the risk of spontaneous or accidental fire outbreaks, narrows harvest windows (especially in humid climates), and can result in extensive contamination of the biomass with soil from field drying operations. If weather conditions are not ideal there can be substantial biomass losses during field drying, and the costs of collection increase. Wet storage systems can reduce these concerns and may also serve as an avenue for *in situ* pretreatment of the biomass to enhance downstream biofuel fermentation processes (Linden et al., 1987; Richard et al., 2001). With a mechanism similar to dilute acid pretreatment, organic acids produced during ensiled wet storage could serve as long-term, mildly acidic, low temperature pretreatment. Pretreatment has remained the most expensive step in the processing of cellulosic feedstock to biofuels and accounts for at least 20% and usually about a third of the total processing cost in most technoeconomic analyses (Wyman, 1999; Yang and Wyman, 2008; Brown and Brown, 2014; Eggeman and Elander, 2015). As a major cost component of biofuel production, any reduction in pretreatment requirements is likely to have commercial value. A feedstock delivery model by Darku (2013) showed that at storage moisture levels < 40% the benefits of wet storage can result in feedstock delivery costs that are lower than dry storage, even considering the costs of transporting the water in the wet biomass.

Previous studies on wet storage have had inconsistent outcomes. Although most reported a favorable impact on downstream processing with reference to the controls (Thomsen et al., 2008; Wendt et al., 2018), some results in some studies show no impact depending on the feedstock or treatment (Linden et al., 1987; Chen et al., 2007; Zheng et al., 2012). However, in most prior research studies the biomass samples were microbially, enzymatically, or chemically treated to enhance storage, and few have looked at the natural ensilage process. In one study without additives, Thomsen et al. (2008) investigated ensiled wet storage using whole-crop maize silage for ethanol production. Although their results failed to demonstrate a pretreatment effect from ensilage, they did show that subsequent pretreatment sugar yields as well as ethanol yield were remarkably improved as a result of the ensilage process. However, the widespread applicability of this result could be confounded by the high starch content of the whole crop (grain and stover) maize feedstock. The differences among wet storage outcomes are dependent, among other factors, on feedstock type. Very few wet storage studies have investigated corn stover, which is the most abundant agricultural residue in the US. Most importantly, none of these previous studies explicitly analyzed the impact of the organic acids produced during ensiled storage, or any modifications of these storage acids during pretreatment and fermentation. In a number of these studies, the feedstocks were washed before subsequent processing, perhaps to prevent interference of the acids with the downstream process. The cost of such washing and the associated wastewater treatment would be hard to justify at a large commercial scale.

Although wet storage of biomass has potential benefits for downstream processing to biofuels, it is also known that most storage acids, including lactic acid, and acetic acid, can under some circumstances inhibit microbial activities including both metabolism and growth (Lund and Eklund, 2000; Deublein and Steinhauser, 2008) and hence negatively affect biofuel fermentations. The impact of such inhibition is dependent on the specific inhibitory compound, its concentration, and also the fermentation organism and conditions used for biofuel production. Although no prior investigations were found on the impacts of silage organic acids on biofuel production, their function in ensilage is to inhibit microbial degradation, and there are ample examples in the food industry of organic acids preserving food through organic acid inhibition or other antimicrobial effects (Lund and Eklund, 2000). Natural acid fermentations are used to preserve sauerkraut, pickles, yogurt, and silage, but unlike foods which will be digested in a mammalian gut, when silage is used as a biofuel feedstock this acidic condition could serve as a potential impediment to downstream fermentations to ethanol and other biofuels and biochemicals. There are a number of reports on negative effects of organic acids with specific reference to ethanol-producing microbes (Palmqvist et al., 1996; Koegel et al., 1997; Zaldivar and Ingram, 1999; Palmqvist and Hahn-Hägerdal, 2000; Klinke et al., 2004; Knauf and Kraus, 2006). Several of these studies focus on the yeast *Saccharomyces cerevisiae*, which is the most common microbe used in ethanol fermentation. These studies showed the inhibitory effect of organic acids on ethanol-producing microbes is dependent on the fermentation conditions, especially initial pH, extracellular-intracellular pH gradient, temperature, the presence of other chemicals, and the type and amount of organic acid present in both dissociated and undissociated forms. Importantly, Taherzadeh et al. (1997), Thomas et al. (2002), and Torija et al. (2003) observed that the effect of organic acids at low levels can sometimes be positive, stimulating growth of fermentative microbes, and ethanol production, and may be necessary for fermentation to proceed. For instance, Taherzadeh et al. (1997) observed that acetic acid could stimulate ethanol production during glucose fermentation at concentrations lower than 10 g L^−1^, or 5 g L^−1^ of the undissociated form at pH 4.5. Torija et al. (2003) also observed that organic acids commonly present in grapes were responsible for the completion of the fermentation process as well as enhanced ethanol yields; none of the controls (i.e., without organic acid of any sort) were able to ferment all the sugars within 21 days.

Other studies have shown that the stimulating and inhibitory effects of organic acids are not restricted to ethanol fermentation. Organic acids can also stimulate butanol fermentation at low concentrations but can be inhibitory above certain threshold concentrations (Cho et al., 2009; Wang et al., 2011; Zhou et al., 2018). Currently, ethanol, and butanol are the only liquid biofuels produced biochemically at a commercial scale by living microorganisms. Enzyme-catalyzed biodiesel production is considered a biochemical process but does not use living organisms, nor do other green drop-in fuels that are produced through hydro/thermo-chemical processes. Because *Saccharomyces cerevisiae* is by far the most common biofuel fermenter in commercial use, it is the focus of the current study.

The main aim of this research was to investigate the individual and cumulative positive and/or negative effects of the organic acids produced during wet storage of corn stover. Specifically, we investigate how these acids interact with the hot water pretreatment process, the potential for reduced severity pretreatment, as well as their effect on ethanol yields. Pretreatment severity is a function of temperature and time (Chum et al., 1990) and reducing either of these would have a corresponding reduction in both capital and operating costs. Any observed potential for reduced severity pretreatment after wet storage would be an indirect measure of the upstream pretreatment capability of ensilage, where the organic acids produced during storage interact with structural bonds in feedstock throughout the storage process. During pretreatment the potential for reduced severity could also result from the interaction of these organic acids, acting as catalyst, with structural bonds during the pretreatment process. Acetic acid and other organic acids generated during hydrothermal pretreatment are recognized as catalyzing agents in enhancing water ionization and cleavage of acetyl group/hydrolysis of hemicellulose (Mosier et al., 2005a; Mohammad, 2008; Zheng et al., 2009). Organic acids, when compared to dilute inorganic acids (as used in “dilute acid pretreatment”), can minimize degradation of hydrolyzed sugars to inhibitors that can impact on subsequent ethanol yield. This has motivated investigations into the use of organic acids (e.g., acetic, lactic, formic, and maleic) as catalysts in hydrothermal pretreatment (Kootstra et al., 2009; Xu et al., 2009; Marzialetti et al., 2011). Organic acid interactions with post-storage pretreatment could therefore be positive.

However, as noted earlier in this introduction, these acids can interfere with the fermentation process. The downstream effect of these acids was determined by examining the inhibitory nature of organic acids on ethanol fermentation by *Saccharomyces cerevisiae*. The use of unensiled, washed, and unwashed silage with and without liquid hot water (LHW) pretreatment extracts provides evidence of the transformation dynamics of these organic acids during common biofuel unit operations, as well as the direct effect of the acids on ethanol fermentation.

## Materials and Methods

### Stover Description and Storage

Corn stover, Pioneer brand 34A20, was obtained from the U.S. Department of Energy's Idaho National Lab. The stover was harvested from the Boyd plot near Boone, IA and field-dried, raked, baled, transported to Idaho, and stored indoors with a tarp cover to prevent dust accumulation. Particle size was reduced to 1” minus (less or equal to 25.4 mm) before storage.

The corn stover had an initial air-dried moisture content of about 7% and was adjusted to six different moisture levels (25, 35, 45, 55, 65, and 75% wet basis) to initiate these wet storage experiments. The use of dry feedstock was to achieve better control and explore well defined feedstock moisture ranges in a controlled experimental environment. Tanjore et al. (2012) showed that the major change resulting from oven drying feedstock is the loss of water-soluble carbohydrates (WSC). In that study the higher WSC available in fresh silage was beneficial in generating a lower storage pH than for the rewetted dry stover, but the rewetted dry stover still achieved an acceptable pH range for effective ensiled storage. Alternatives to drying and rewetting, such as harvesting as drying occurs in the field over several weeks during plant senescence, would create its own set of issues. A difference in stover harvest date of even 2 weeks has been shown to have great influence on silage response and composition (Russell, 1986). In the present study moisture adjustment was accomplished by spraying with an appropriate amount of water, covering with plastic, and leaving the samples overnight for the moisture to be thoroughly absorbed into the fibers. The moisture adjustment was within ±2 percentage units of the target moisture level. For each adjusted moisture level, there are corresponding samples that were not ensiled and used as control (Day 0).

Corn stover was stored at a dry bulk density of about 160 Kg/m^3^ in 470 ml glass jars that were tightly sealed to create anaerobic conditions. This density provides sufficient compaction to facilitate the silage process and is also comparable to corn stover dry bale densities and the average density used in conventional dry storage systems for hay. Storage duration was 220 days at two temperatures: ambient, which was ~23 ± 1°C, and 37°C. The higher 37°C temperature, which is observed in warm climates, accelerates fermentation. This allows for shorter experiments and also stresses some of the microorganisms. Higher temperatures, non-optimal moisture or substrate conditions can also encourage secondary fermentations from organisms such as clostridia which can reduce silage quality (Weinberg et al., 2001). Including the 37°C treatment thus accomplishes several goals: to subject the silage process to this temperature stress, to create wide variations in silage outcomes, and to then observe how that wide variations in silage outcomes impact downstream processing.

Experiments were performed in triplicate. After storage, samples were dried in a HotPack convection oven at 55°C, ground using a 2 mm screen on a Wiley Mill (Model 4, Thomas Scientific, Swedesboro, NJ) and stored at room temperature in sterile airtight Whirl-Pak bags (Nasco, Fort Atkinson, Wisc.) Prior to pretreatment, replicates from each storage condition were thoroughly mixed together to reduce variability among replicates before resampling. Composition of the feedstock before and after storage was measured in accordance with the NREL standard protocols with the exception that the feedstock drying temperature was 55°C (Hames et al., 2008; Sluiter et al., 2008a,b,c). At this drying temperature volatilization of acids and alcohols was expected to be small, other than possibly methanol, or ethanol. However, these alcohols were not expected to be present in the ensiled biomass at levels that might be inhibitory to *Saccharomyces cerevisiae* (Driehuis and van Wikselaar, 2000).

### Organic Acid Measurements and Pretreatment

Collection of soluble extracts and measurements of pH and organic acids of feedstock were performed before and after storage. Samples were thoroughly mixed before sub-sampling, and deionized water was added at a ratio of 1:10, i.e., 5 g of sample to 50 ml of water. The mixtures were shaken for 30 min at 200 rpm using a Barnstead SHKA 2000 open air platform shaker (Barnstead International, Dubuque, IA) after which the extracts were filtered through Whatman No.1 paper. The pH of storage extracts was determined using a pH meter (SevenEasy S20, Mettler-Toledo International Inc, Columbus, OH). The collected extracts were filtered again using 0.2 μm PTFE filters, diluted 20-fold and analyzed using Ion Exclusion Chromatography System (Dionex ICS 3000, Thermo Fisher Scientific Inc., Sunnyvale, CA) for types and amount of organic acids. Separation was performed at 30°C using IonPac ICE-AS1 guard (4 × 50 mm) and analytical (4 × 250 mm) columns with 100 mM methanesulfonic acid eluent at a flow rate of 0.16 mL/min. Organic acids were detected with a photodiode array detector (Dionex UVD 340U, Thermo Fisher Scientific Inc., Sunnyvale, CA) at a wavelength of 210 nm. Thirteen different potential acids (lactic, acetic, butyric, pyruvate, isobutyric, valeric, isovaleric, propionic, tartaric, malic, formic, citric, succinic) were used as standards.

The impact of organic acids on liquid hot water (LHW) pretreatment requirements was investigated using washed and unwashed samples of dry ground ensiled (Day 220) and unensiled (Day 0) corn stover. Ensiled samples are the moisture adjusted stover stored under anaerobic conditions for 220 days, while the unensiled samples are the corresponding Day zero samples. Washed samples were washed with deionized water using an Accelerated Solvent Extraction (ASE) system (ASE 350, Thermo Fisher Scientific Inc., Dionex ASE 350, Sunnyvale, CA) set at 40°C with three static cycles of 10 min each, 100% flush, and a purge time of 200 s for 66 ml cells. The purpose of washing was to remove all organic acids produced during storage in order to prevent any involvement or interaction with the pretreatment procedure. In this way, the washed samples served as the control against which unwashed samples were compared to assess the impact of organic acids on the pretreatment process. Washed samples also provided controls to understand whether any change in pretreatment outcome is as a result of acid interaction during storage or acid interaction during pretreatment, by comparison with unwashed samples of unensiled and ensiled stover, respectively. Only 37°C samples were washed for comparison.

Liquid hot water (LHW) pretreatment of samples was also accomplished using the ASE 350 equipment, with each sample replicated four times. LHW pretreatment is a well-established and effective strategy that involves heating water-saturated or moist feedstock at high temperatures (160–220°C) under high pressure to maintain the liquid state for a few minutes, without any chemical additives. Optimum conditions reported by Mosier et al. (2005b) for controlled pH LHW pretreatment were 190°C for 15 min, and these conditions were used as the benchmark for reduced severity comparisons. This standard pretreatment condition with the ASE was defined as 190°C, 1 static cycle of 15 min, 0% flush volume and a purge time of 120 s for 10 ml cells, using deionized water as the solvent. Each 10 ml ASE cell was filled with 1.5 dry gram of sample. Solids loading was 14–20% and 13–15% for unwashed and washed samples, respectively. The solids loading is the percentage of dry solids to total liquids after pretreatment. The variability in solids loading is subject to the amount of water added by the ASE 350 during the filling and heating stage. At the end of the retention time, the liquid is purged out along with some other soluble and insoluble components and is described as pretreatment extract. The pH and organic acid composition of the pretreatment extract were determined using the same equipment and methodology used in storage extract described above. The potential for reduced severity pretreatment was investigated by comparing shorter retention times (5 and 10 min) with the standard 15 min, all at 190°C.

Pretreatment extracts, 500 μl each, were diluted 30-fold and filtered through 0.2 μm PTFE syringe filters prior to analyzing for organic acids and inhibitors caused by sugar degradation (5 -Hydroxymethyl furfural (HMF) and furfural), again using the Dionex ICS 3000 for ion exclusion chromatography. Separation and detection of organic acids followed the same method described above for the before and after storage samples. Inhibitors were also detected with the same photodiode array detector but at wavelengths of 270 nm.

Xylan and glucan removal during pretreatment was also determined. Xylan removal, a proxy for hemicellulose hydrolysis, was used as a comparative indicator of the relative effectiveness of the different pretreatment conditions. To measure the removal of these sugar polymers, the pH of the undiluted extracts was first measured. The pretreatment extracts had pH levels >3.5 but <5. For this pH range, the hydrogen ion concentrations had significant digits at the 4th or 5th decimal place. As a result, volume of acid required for the monosaccharide assay was practically the same. Based on the pH range, 52.3 μl of 72% w/w sulfuric acid (Sigma–Aldrich, St. Louis, MO) was added to 1.5 ml of each extract to obtain a final concentration of 4% sulfuric acid in 10-ml autoclave safe bottles. Bottles were tightly covered using rubber stoppers with crimped aluminum seals and placed in autoclave, together with sugar recovery standards, at 121°C liquid setting for 1 h. The acid-hydrolyzed extracts were filtered through 0.2 μm PTFE filters and diluted 400-fold. Monosaccharide composition was determined using Dionex ICS 3000 ion exclusion chromatography. Separation was by high pH anion exchange at 30°C using CarboPac PA20 guard (3 × 30 mm) and analytical (3 × 150 mm) columns with 2 mM sodium hydroxide (NaOH) eluent at a flow rate of 0.5 ml/min. Detection of the monosaccharides was by pulsed amperometric [electrochemical] detection at gold working electrodes, using a quadruple waveform. Xylan and glucan removal were calculated from xylose and glucose concentrations, using conversion factors of 0.88 and 0.90, respectively. Equation 1 was used in calculating xylan removal. For glucan removal, the various xylan parameters were replaced by the relevant glucan parameters.

(1) $$\begin{array}{r} \%\;Xylan\;removed=\frac{x_{p\;}\times 0.88}{\frac{\left(X_{DM}-\;x_{s}\times 0.88\right)}{100}\times DM}\times 100 \end{array}$$

Where

*x*_*p*_ = Mass of xylose in pretreatment extract (g)

*x*_*s*_ = Xylose degraded during storage (% dry matter)

X_DM_ = Original xylan content of corn stover before storage (% dry matter)

DM = Dry Matter (Here as dry mass of corn stover that was pretreated) (g).

### Simultaneous Fermentation and Saccharification

After pretreatment, the solids content of each pretreatment cell was directly transferred to a 50-ml centrifuge tube for fermentation. Pretreatment extracts were collected separately during the extraction process. For each storage condition investigated, two replicates were fermented with pretreatment extract, and two without extract. The pretreatment extract contains most of the inhibitory compounds, which are soluble. Thus, there were two steps in the overall process when inhibitors could be separated, first when washing samples after wet storage but before pretreatment, and second when extracting liquids after pretreatment. These two separations (or their absence, for the “unwashed” storage samples and “with extract” fermentation treatments) make it possible to determine the impact of organic acids and other inhibitors formed during storage and/or pretreatment separately with respect their contribution to fermentation inhibition. Although the wet storage organic acid profile may transform during pretreatment, the new post-pretreatment organic acid profile is assumed to be influenced by, or a product of, the acids produced during storage.

Simultaneous fermentation and saccharification (SSF) was carried out in tightly sealed 50-ml centrifuge tubes. Samples fermented with pretreatment extract had a solids loading of 8.4% ± 0.1%, while washed and unwashed samples fermented without extract had a solids loading of 5.2% ± 0.1%. The solids loading was calculated as the ratio of dry mass of feedstock used in fermentation to the mass of total fermentation liquids. For samples without extract, some solids were lost in the pretreatment extract resulting in the lower solids loading. The fermentation broth contained the following components prepared in a cocktail before addition: Penicillin-Streptomycin at a final concentration of 30 μg/mL (0.1% v/v) to prevent bacterial growth; citric acid buffer (pH 4.5) at 0.05M to maintain a pH of 4.8, which is in the optimum range for enzyme activity; Yeast peptone (YP) as a microbial nutrient at 1% broth volume; commercial cellulase (Spezyme CP, Genencor, Rochester, NY) at 15 filter paper units (FPU)/g glucan complemented with a commercial β-glucosidase (Novozyme 188, Novozymes A/S, Bagsvaerd, Denmark) at 60 cellobiase units (CBU)/g glucan. The microorganism used for fermentation, *Saccharomyces cerevisiae* NRRL Y-2034, was obtained from the USDA ARS culture [NRRL] collection. *Saccharomyces cerevisiae* Y-2034 is a wild type 6-carbon sugar fermenting yeast. The yeast was grown in YPD media (10 g/L yeast extract, 20 g/L peptone, and 50 g/L dextrose) for about 24 h after which the cells were centrifuged at 4,200 rpm for 5 min. The supernatant was discarded, and the cells were washed in 1 × PBS (Phosphate Buffer Solution: 138 mM sodium chloride, 2.7 mM potassium chloride, 12 mM sodium and potassium phosphates, pH 7.4). After washing, cells were resuspended in PBS and used as fermentation inoculant. Each tube was inoculated with appropriate volume of inoculant to obtain an initial OD_600_ of 0.5. Fermentation tubes were vortexed for ~5 s to mix contents before incubation for 72 h. The fermentation temperature and agitation, 35°C, and 110 rpm, were achieved using a lateral motion hot water bath. However, the vertical placement of tubes in the bath did not provide the complete mixing intended by the agitation. Tubes were therefore removed twice (every 24 h) within the fermenting period and inverted twice to mix contents. Control samples included enzyme-yeast blanks and Avicel (α-cellulose). At the end of the fermentation period, samples were centrifuged, and the supernatant collected in micro-centrifuge tubes. The supernatant from each fermentation broth was diluted 9-fold and analyzed for ethanol using the YSI 2700 SELECT™ biochemical analyzer (YSI Inc., Yellow Springs, OH) with 2% precision.

### Data Analysis

Results were analyzed using statistical tools such as analysis of variance (ANOVA), Tukey's multiple comparison test, and regression analysis. All statistical tests were conducted using Minitab 14 (Minitab Inc., State College, PA) at a significance level, α, of 0.05. Results are reported in most cases as means along with the standard deviation of mean (±) as a measure of variability.

## Results and Discussion

### Pretreatment pH

After pretreatment the pH of the biomass feedstock generally decreased relative to the pH before pretreatment. This result was expected, and can be attributed to deacetylation of xylan, which is the main component of herbaceous hemicellulose [68–72% in this study], at high temperatures leading to the formation of acetic acid (Zhou et al., 2010; Johnson et al., 2017). This acid in turn interacts with the pretreatment process by serving as hydrolytic catalyst, providing free protons. Since pH is an indication of hydrogen ion concentrations, the change in pH can thus indirectly indicate LHW pretreatment activity. Compared to unensiled stover, smaller differences were observed between pH after wet storage and subsequent pretreatment pH of unwashed ensiled feedstock. The average difference for unensiled feedstock was 2.2 pH units while feedstock ensiled at 23°C and 37°C had average differences of 0.08 and 0.18 pH units, respectively. Each one-unit difference in the pH corresponds to a 10-fold change in acidity or hydrogen ion concentration. Two factors—hemicellulose degradation during storage and the buffering capacity of organic acids—are likely responsible for the smaller differences in ensiled feedstock. It was observed that on average, 10% of hemicelluloses were degraded after 220 days of storage, mainly through xylan and acetyl degradation. The acetyl groups constitute 3.8% to 5% of the stover total dry matter and 12–15% of the hemicellulose fraction, and are the most susceptible components to the low temperature acid hydrolysis that occurs during ensilage. This susceptibility was evident from the large amount of the acetyl fraction degraded during storage, up to 49.47% ± 3.12 at 35% moisture, 37°C (see Supplementary Material), with a maximum of ~56%. This implies that fewer acetyl groups would be available in ensiled feedstock for conversion to acetic acid during pretreatment. Alternatively, the organic acids present in ensiled samples, up to 9.1% of total dry matter compared to < 0.5% for unensiled samples, could serve as buffering agents, resisting pH change. This second factor is supported by the larger pH change in washed ensiled samples compared to unwashed samples. Without storage derived organic acids interfering with pretreatment, the decrease in storage pH of 0.34 mean pH units during pretreatment of washed samples was more than double that of the unwashed ensiled stover but still much smaller than that of unensiled feedstock. Assuming deacetylation is the main factor accounting for change in pH, the results from this study suggest that the theoretical maximum number of hydrogen ions that can be released from acetyl component of corn stover during storage and/or pretreatment may be enough to bring down the pH to a vicinity of pH 4. On average, the pH of washed ensiled samples (pH 4.08) was lower than unwashed samples (pH 4.24) (*p* = 0.001).

Although the pH of unensiled samples decreased more dramatically during pretreatment, mean resultant pH values were still higher (4.44 ± 0.17) than for the ensiled samples (4.26 ± 0.16) (*p* < 0.0001). Relating this to acetyl content, unensiled stoved had 128% more acetyl than ensiled stover, if using 56% upper limit degradation during storage. If all these acetyl groups in unensiled samples were removed during pretreatment, that would imply about 2.28 times the hydrogen ions compared to ensiled samples. From Table 1, it can be inferred that this drastic decrease in pH was a result of more acetyl in the unensiled feedstock, which was then available for hydrolysis to acetic acid during pretreatment. In general, pH decreased with increased pretreatment time. For unensiled samples the pH values at all three-time levels (5, 10, 15, min) were significantly different from each other (4.64 ± 0.09, 4.42 ± 0.08, 4.27 ± 0.06, respectively; *p* < 0.0001) and feedstock moisture had no significant impact. For ensiled samples, there was no significant difference between 10 and 15 min (4.23 ± 0.10 and 4.18 ± 0.16), both of which were lower than 5 min (4.37 ±0.14) (*p* < 0.0001). With respect to storage moisture, there was no significant difference in pH at all moisture levels except for 25% moisture, which was higher than the 45% and 55% moisture treatments (*p* < 0.0001). There was also no significant impact of storage temperature on pretreatment pH. In all, the pH values were moderate and conducive to both enzymatic hydrolysis and ethanol fermentation.

**Table 1:** Relating hydrogen ion concentration to acetyl group hydrolysis during pretreatment.

	**Before pretreatment**		**After pretreatment**		**Hydrogen ions from pretreatment ([H^**+**^]_**after**_-[H^**+**^]_**before**_)**
	**pH**	**[H**^**+**^**]**	**pH**	**[H**^**+**^**]**	
Ensiled^*^	4.38	4.16869E-05	4.26	5.49541E-05	1.32671E-05
Unensiled	6.67	2.13796E-07	4.44	3.63078E-05	3.6094E-05
Ratio ([H^+^]_Unensiled_ /[H^+^]_Ensiled_)					2.72
**Acetyl content (%)**	**Unensiled**		**Ensiled**		**Ratio unensiled:ensiled**
High end degradation (35% moisture, 37°C)	5.07		2.22		2.28
Low level degradation (45% moisture, 23°C)	4.66		4.62		1.01
Mean across moisture at 37°C	4.34		2.53		1.72
Mean across moisture at 23°C	4.34		3.98		1.09
Max. Expected ratio^**^ ([H^+^]_Unensiled_ /[H^+^]_Ensiled_)					2.28

* The pH of ensiled and unensiled stover is mean pH of all sample without regards to moisture levels or temperature.

** Expected ratio if all acetyl in sample is completely hydrolyzed to hydrogen ions.

*Shaded values highlights mean ratio of hydrogen ion concentration in pretreated extract and expected ratio form complete degradation of acetyl group (unensiled:ensiled)*.

### Glucan and Xylan Removal

Glucan removal during pretreatment in unensiled stover was ~58% higher than ensiled (4.49% ± 0.65 vs. 2.84% ± 0.56; *p* < 0.0001) and storage temperature had no significant impact on amount removed (*p* = 0.157). Pretreatment time also had no significant impact on glucan removal (*p* = 0.742 ensiled, 0.525 unensiled). Similarly, xylan removal from ensiled stover was not significantly different across the various pretreatment times (*p* = 0.210), with the results indicating 5 min (27.67% ± 3.29) was just as effective as 15 min (28.68% ± 2.08). This result may have important commercial implications if the assumption that xylan removal reflects the extent of pretreatment is valid. In contrast, xylan removal from unensiled stover was significantly lower after 5 min of pretreatment (22.23% ± 2.06) compared to 10 (26.62% ± 2.31) and 15 min (27.30% ± 1.12) of pretreatment (*p* < 0.001). At the longest retention time of 15 min, xylan removals for ensiled and unensiled stovers were not significantly different from each other. This result suggests as retention time or pretreatment severity increases wet storage benefits for pretreatment are masked. Ensiled stover had significantly higher xylan removal, about 28% on average, compared to 25% for unensiled samples (*p* < 0.0001). The minimum xylan removed was 17% and maximum was 34% for unwashed samples.

When considering the effect of storage moisture content on glucan removal, the results indicated samples ensiled at 45–75% moisture and subsequently pretreated were not significantly different from each other, while glucan removal was higher in samples ensiled at 25% and 35% moisture. For unensiled samples, pretreatment of samples in the range of 25% to 55% moisture did not experience significantly different glucan removal. With respect to xylan removal, the effect of moisture was only observed in the ensiled samples. At 23°C, xylan removal was highest at 35% moisture (30.84% ± 2.48) although this difference was only significant relative to the 25% and 65% moisture samples, and both of these treatment conditions were not significantly different from other moisture levels (*p* = 0.007). At 37°C, xylan removal at 45%, and 55% moisture was only significantly higher than the 35% moisture treatment. These results showed storage temperature had some effect on xylan removal and revealed a significant interaction with storage moisture (*p* < 0.0001). Generally, samples stored at 23°C experienced more xylan removal during pretreatment than samples stored at 37°C (27.58% ± 3.17 vs. 25.05% ± 3.21; *p* = 0.001). The lower average xylan removal rate after ensilage at 37°C could be biased by a few samples at the extremes of the storage moisture range, which had no lactic acid or lower lactic acid at 37°C compared to 23°C.

Washing ensiled samples appeared to increase xylan removal. For example, during pretreatment for 15 min the washed ensiled samples experienced more xylan removal than the unwashed ensiled samples, 36.36% ± 4.56, and 23.89% ± 2.73, respectively (*p* < 0.0001). Since washed samples do not contain organic acids, the implication is that contribution of silage organic acids to pretreatment is primarily during the storage process, rather than during the subsequent conventional pretreatment process. Although organic acids accelerate xylan removal during pretreatment, they may also interfere with, and limit xylan removal during conventional pretreatment. This acceleration and limitation was observed in data on the amount of xylan removed in 5 min compared to 15 min in unwashed stover. Under these circumstances xylan removal did not increase significantly with pretreatment time from 5 to 15 min, although higher removal from the washed samples indicated more xylan was potentially available for removal in unwashed samples. Despite this apparent interference, xylan removal in wet storage samples was still better [at shorter retention times] or comparable [at longer retention times] to unensiled samples, as previously discussed.

### Organic Acids and Inhibitors From Pretreatment

#### Organic Acids

**Figure 1:**
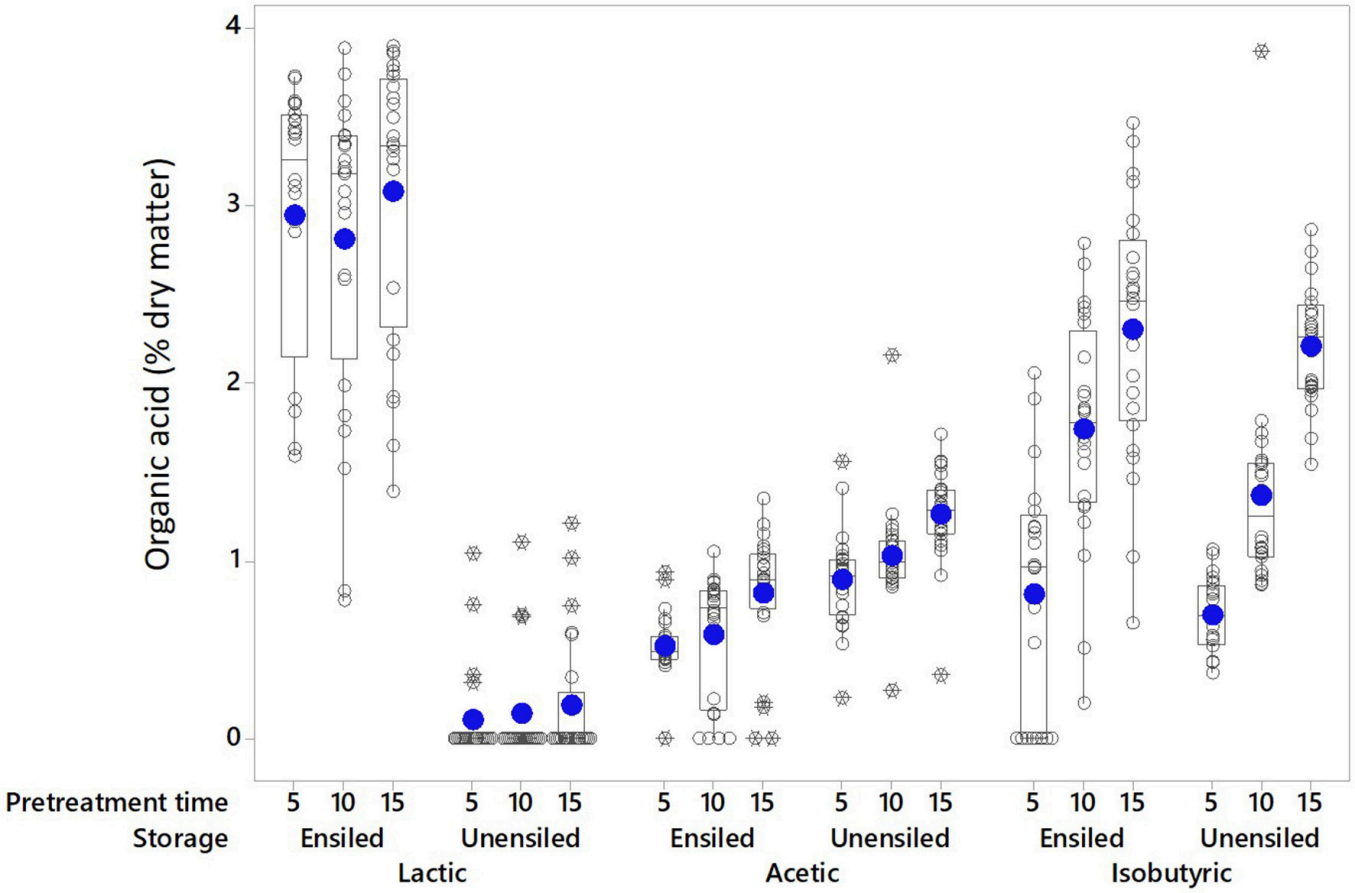
The main pretreatment acids in extracts of unwashed stover are shown at the various pretreatment retention times without regard to moisture levels. Solid circle = Mean; open circle = Individual values; Rectangle = Interquartile range; X = Outliers (extreme deviation from other observations; identified and calculated in the boxplot graphing tool as at least 1.5 interquartile ranges from edge of box).

**Figure 2:**
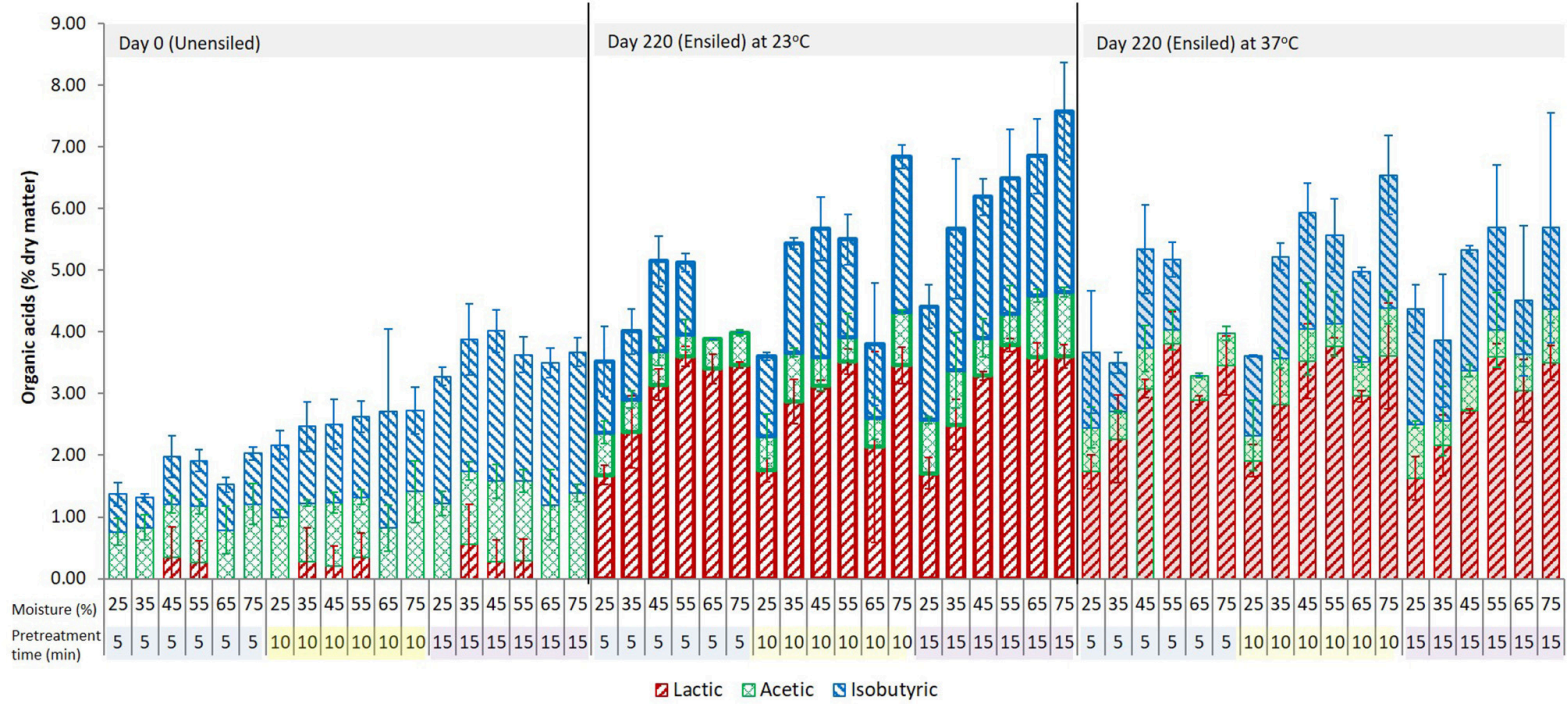
The three dominant acids in the pretreatment extract from unwashed ensiled stover at various storage moisture levels and pretreatment retention times. Error bars are ± standard deviation of mean; *n* = 4 per treatment group.

Organic acids are a product of anaerobic fermentation and provide the primary mechanism of preservation during ensilage; some can also be formed during pretreatment. The main acids identified in the pretreatment extracts of unwashed corn stover feedstock were lactic (≤4.0% DM), acetic (≤2.2% DM), and isobutyric (≤3.9% DM) acids (see Figures 1, 2). Individually, none of these acids exceeded 6 g L^−1^ (mass per pretreated volume). Low levels of tartaric, malic, formic, pyruvic were also detected. Wet storage and pretreatment conditions affected which acids were dominant, and these were different for different conditions. Lactic acid was the dominant acid in wet stored, pretreated feedstock (2.94% DM ± 0.81 [Ensiled] vs. 0.14% DM ± 0.31 [Unensiled]) while acetic acid was dominant in unensiled samples [1.06% DM ± 0.31 [Unensiled] vs. 0.64% DM ± 0.33 [Ensiled]]. Isobutyric acid was equally high after pretreatment for both before and after storage samples (1.61% DM ± 0.91 [Ensiled] vs. 1.42% DM ± 0.75 [Unensiled]).

For most ensiled treatments, the amount of lactic acid increased during pretreatment while the amount of acetic acid decreased. Samples with lower lactic acid (0.0 < 1.50% DM; 25% and 75% moisture) prior to pretreatment generated more lactic acid (up to 4.22% DM) and samples with higher lactic acid (1.94–3.20% DM) generated < 1.40%, with ~68% from this group having ≤ 0.7% above storage levels and 17% showing a slight decrease below storage levels (see Supplementary Data). Lactic acid could have been produced through hydrothermal deamination and hydroxylation of amino acids, or in small amounts via hydrothermal degradation of polysaccharides (Quitain et al., 2002; He et al., 2008). Another potential source of lactic acid generation may be a reaction of acetic acid formed during storage with formaldehyde, which is also produced during pretreatment (see Equation 2).

$$\begin{array}{r} C_{2}H_{4}O_{2\;}\;+\;CH_{2}O\;\to \;C_{3}H_{6}0_{3} \end{array}$$

(2) $$\begin{array}{r} \text{Acetic acid }+\text{ Formaldehyde }\to \text{ Lactic acid} \end{array}$$

At standard conditions, this reaction is spontaneous. This pathway is proposed based on the disappearance, during pretreatment, of some of the acetic acid present after wet storage. Also, compared to washed samples with no storage acids, hence no acetic acid, the amount of lactic acid generated during pretreatment was less than the amount produced during storage but washed out before pretreatment. Lactic acid in the pretreatment extract of washed samples was in general <0.5% DM. Formaldehyde, assumed in Equation 2 as reacting with acetic acid, can be produced from thermohydrolytic degradation of xylose (Schäfer and Roffael, 2000; Roffael and Hüster, 2012).

About half of the unwashed wet storage samples with an acetic acid concentration lower than 1% DM [mostly samples from 25 to 45% moisture] had nearly a percentage point decrease (up to 0.9% DM) in the original amount after pretreatment, while the other half showed an increase (up to 0.8% DM) above the wet storage amount. In contrast, wet storage samples with acetic acid concentrations >1% to 2.8% DM [55–75% moisture] showed acetic acid decrease, which was up to 2.3% of the dry matter in the higher acetic acid samples. Isobutyric acid in pretreatment extracts was high and was not significantly different for both unensiled and ensiled samples. In most cases, there was an increase above the initial wet storage amount (up to 3.1% DM) except for the retention time of 5 min, where 65 and 75% moisture samples showed a decrease in isobutyric acid.

In the pretreatment extracts from washed samples, malic acid was dominant (2.11% ± 1.73 DM), far more than acetic and lactic acids which averaged ≤1% DM and ≤0.51% DM, respectively. Formic acid amounts were comparable to lactic acid. Traces of succinic acid were observed in the 75% moisture samples. No isobutyric acid was present in pretreatment extracts from any washed samples. This lack of isobutyric acid in washed samples contrasts with the relatively high levels of isobutyric acid after pretreatment in the unwashed samples. The increase in the isobutyric acid concentration of unwashed samples could thus be due to interactions of organic acids or other extractives washed out of the feedstock. Unlike acetic and lactic acids, isobutyric acid is usually not reported in studies of hydrothermal processing of lignocellulosic materials, and a specific abiotic reaction that could occur during pretreatment has not been elucidated.

Total organic acid in pretreatment extracts were not significantly different at the various pretreatment retention times (*p* = 0.353) and various storage moisture levels (*p* = 0.306). However, wet storage did have an impact. Organic acids were virtually absent in control (Unensiled) samples prior to pretreatment. These unwashed unensiled samples behaved similarly to the washed wet stored samples, generating more acids during pretreatment than unwashed wet stored samples. On average, however, total acids from pretreated unwashed wet stored feedstock were still higher (~6.54% DM) than for unwashed unensiled samples (~4.46% DM) (*p* < 0.0001).

The unwashed samples also showed apparent changes in the organic acid profile after pretreatment. Butyric acid, present in a number of unwashed high moisture samples in amounts >1% DM, disappeared completely. Individually, the amounts of lactic acid and acetic acid in ensiled samples were not significantly different at the various pretreatment times (*p* = 0.405 and 0.118, respectively). For unensiled samples, the lactic acid concentration was not different for the various pretreatment times (*p* = 0.642). However, the acetic acid concentration at 15 min was significantly higher than at 5 and 10 min (*p* < 0.0001). With respect to temperature, samples stored at 23°C on the whole had lower lactic acid (*p* = 0.001) but more acetic (*p* = 0.002) than 37°C samples after pretreatment. Based on the amount of storage acetic and lactic acids at 23°C and 37°C, a similar inverse relation was observed as was described previously, in which samples with relatively lower initial [storage] acids had higher amounts generated during pretreatment than samples with higher initial [storage] acids and vice-versa.

#### Inhibitors

In addition to organic acids, two sugar degradation products 5-(Hydroxymethyl) furfural (HMF), and furfural were measured in pretreatment extracts. The former derives from hexoses and the later from pentoses, and both are known to inhibit many ethanologens including yeast. Generally, both of these inhibitors were higher in ensiled samples compared to unensiled samples and both were not affected by storage temperature (*p* > 0.5). On average, HMF concentrations were <0.05% DM (0.039% ± 0.018) and furfural concentrations were <0.5% DM (0.47% ± 0.27) in unwashed ensiled samples. These HMF and furfural concentrations were about 30 and 75% higher, respectively, than the amounts in unensiled samples. The ratio of xylan removed to glucan removed was approximately the same as the ratio of furfural to HMF produced, and this ratio was on the order of 10. On a per [pretreated] volume basis, concentration of HMF and furfural were 0.03 ± 0.05 g L^−1^ extract and 0.48 ± 0.32 g L^−1^ extract, respectively.

In contrast to unwashed samples, washed samples had no HMF in the pretreatment extracts, but far higher furfural concentrations than were observed in the unwashed samples, averaging 1.12% ± 0.26 (DM) or 1.5 g L^−1^ extract at 15 min retention time. Glucan removal during pretreatment from washed samples was higher than that from unwashed samples (3.28% DM ± 0.38% vs. 2.81% DM ± 0.43%, *p* = 0.017). This higher glucan removal was expected to result in larger amounts of HMF in the pretreatment extracts from the washed samples, but this was not the case. This suggests that HMF was produced from degradation of preexisting glucose in the water-soluble components of corn stover or glucose hydrolyzed during wet storage rather than from the glucose produced from structural degradation during the pretreatment process. This result thus supports the hypothesis that decomposing valuable feedstock components to simpler, more bioavailable forms during wet storage increases their risk of being degraded in subsequent processing to less valuable forms. Alternatively, it is possible that other water-soluble compounds in the stover or produced during ensilage may catalyze glucose degradation or serve as reaction partners in the formation of HMF in unwashed samples. Their absence in the washed samples would therefore hinder the formation of HMF. The higher furfural could be attributed to the higher xylan removal from washed samples (*p* < 0.0001). At 15 min pretreatment retention time xylan removal was 36 and 24% of theoretical for washed and unwashed samples, respectively. Furfural generated from the pretreated washed samples previously stored under the extreme moisture conditions (25 and 75%) were lower [<1%-point, g furfural/g biomass] than amounts produced during pretreatment of washed samples from the mid-range 35–65% moisture wet storage samples, which were not significantly different from each other. As noted earlier, furfural is formed when xylose is degraded and from pretreatment extract analysis, samples at these extreme moistures had lower xylan removal, hence lower furfural.

The amounts of both the HMF and furfural inhibitors increased with pretreatment time as expected. For unensiled feedstock, amounts of HMF and furfural generated at each pretreatment retention time were significantly different from each other (*p* < 0.05) (see Table 2). In wet stored unwashed stover, HMF generated during 5 and 10 min of pretreatment were not significantly different and were lower than the amount generated in 15 min. Furfural, however, was different for all pretreatment times. For the unensiled vs. wet stored stover, there was more than a 100 and a 60% increase in furfural, respectively, for every 5 min increase in pretreatment time. Furfural produced during pretreatment was not significantly different for the various storage moistures. HMF was also not significantly different except for 45% moisture ensiled stover samples (which had higher amounts than observed for the 65% samples) and 35% moisture (which had higher amounts than observed for the 75% moisture samples of unensiled stover).

**Table 2:** Furfural and HMF generated during liquid hot water pretreatment of unwashed corn stover, 23°C, averaged across all moisture levels (*N* = 24 per treatment group).

**Pretreatment time (minutes)**	**Furfural (% dry matter)**		**HMF (% dry matter)**	
	**Ensiled**	**Unensiled**	**Ensiled**	**Unensiled**
0	0^†^	0^†^	0^†^	0^†^
5	0.229 ± 0.124	0.079 ± 0.019	0.027 ± 0.016	0.015 ± 0.006
10	0.414 ± 0.080	0.236 ± 0.027	0.036 ± 0.010	0.028 ± 0.007
15	0.670 ± 0.287	0.490 ± 0.062	0.052 ± 0.019	0.046 ± 0.013
*p*-value	<0.0001	<0.001	0.001	<0.001
Regression^*^	0.0479 × *prt time*	0.0289 × *prt time*	0.0038 × *prt time*	0.003 × *prt time*
*R*^2^	~0.84	~0.86	~0.63	~0.72

* All intercepts set to zero.

R^2^ was similar in zeroed and actual intercept except for unensiled furfural in which the actual equation [(0.0411 × pretreatment time)−0.1426] had an R^2^ of ~95%.

† *Analysis of water-soluble extracts collected before pretreatment showed there was no furfural or HMF present in the feedstock before pretreatment*.

### Fermentation

#### Ensiled vs. Unensiled

In most cases, there was no significant difference in ethanol yields of unensiled (Day 0) and ensiled (Day 220) unwashed stover. This was true whether the stover samples were fermented with or without their pretreatment extracts (*p*: with extract = 0.745, without extract = 0.235) (see Table 3). However, the pretreatment response of samples with or without wet storage was different in terms of the dominant acids, inhibitors produced, and xylan removed at shorter retention times. Organic acids, furfural, and HMF were higher in ensiled samples, and so was xylan removal. The first group can adversely affect fermentation yields for organisms such as yeast, depending on their concentrations and the pH of the fermentation broth, while xylan removal could make glucan more accessible, and thus favor ethanol yields. This is especially true in systems like this study, where a typical hexose fermenting yeast was used. Xylan removal may also be beneficial for fermentations with consolidated bioprocessing organisms such as *Clostridium thermocellum*, for which pentoses have been shown to be inhibitory (Verbeke et al., 2017). Ranges of the dominant acids in the fermentation broths, on mass per fermentation volume basis, were 0.00–2.17 g L^−1^, 0.00–1.07 g/L, and 0.00–2.69 g L^−1^ for isobutyric, acetic, and lactic acids, respectively. Acetic acid was dominant in unensiled feedstock while lactic was dominant in ensiled feedstock.

**Table 3:** Ethanol yields, on percent of theoretical basis, averaged for all moisture levels at each different pretreatment retention time^*^.

		**Unensiled (Day 0)**				**Ensiled (Day 220)**			
**Pretreatment time (min)**		**5**	**10**	**15**	***p*****-value**	**5**	**10**	**15**	***p*****-value**
Unwashed	With extract (23°C)	46.65 ± 1.91^a^	50.46 ± 2.10^b^	55.27 ± 1.60^c^	<0.0001	44.00 ± 2.91^a^	49.43 ± 3.65^b^	57.59 ± 6.41^c^	<0.0001
	No extract (23°C)	40.07 ± 1.82^a^	45.45 ± 3.59^b^	49.11 ± 3.71^c^	<0.0001	41.29 ± 8.84^a^	48.11 ± 1.86^b^	50.31 ± 4.89^b^	0.002
	With extract (37°C)					38.58 ± 2.28^a^	41.91 ± 3.16^ab^	46.86 ± 11.29^b^	0.021
Washed	With extract (37°C)					33.25 ± 2.75^a^	43.13 ± 5.37^b^	41.56 ± 6.21^b^	<0.0001
	No extract (37°C)							45.65 ± 6.62	
*P*-value (down)		<0.0001	0.001	<0.0001		<0.0001	<0.0001	<0.0001	

* Mean results along with standard deviation pooled across the six moisture levels.

*Means without a common superscript letter in a row differ significantly as analyzed by two-way ANOVA and the TUKEY test; the same color down column indicates not significantly different. n = 12 per treatment group. Empty cells = no data because these conditions were not part of this study design*.

Acetic acid levels above 0.50 g L^−1^ lead to intracellular accumulation that could affect cell growth, ethanol production or both, while lactic acid concentrations >8.0 g L^−1^ could lead to cell death depending on type of yeast and intracellular pH (Narendranath et al., 2001; Ingledew, 2003). Although lactic acid concentrations in the present study were much lower than this 8 g L^−1^ level, 92% of unensiled samples in this study had acetic acid concentrations >0.50 g L^−1^. However, the fermentation pH of 4.8 in this study is higher than the pH values of 3.0–4.0 in Narendranath et al. (2001) and Ingledew (2003). Thus, the effect of these acids on fermentation microbes in the present study is expected to be less than in these prior studies, due to the lower amount of undissociated acids. A more recent study conducted by Xu et al. (2010) found that acetic acid, which is more inhibitory than lactic acid, only inhibited ethanol yields when the concentration used in LHW pretreatment exceeded 6% DM. In addition, at 6% DM, 0.51 g L^−1^ furfural, 0.07 g L^−1^ HMF, and 4.5 g L^−1^ acetic acid present in fermentation broth did not have any inhibitory effect on fermentation yields but was similar to yields of control samples without acetic acid. When acetic acid used in pretreatment was less than 6% DM, concentrations of ethanol were higher than the control, up to 8.63 g L^−1^ compared to 7.63 g L^−1^ in the control for extracted pretreatment liquor; and up to 33.72 g L^−1^ compared to 21.95 g L^−1^ for the solid fraction. The maximum ethanol yield from the pretreatment liquor was obtained when acetic acid was 1.0%–3.0% DM, equivalent to 1.5 g–2.5 g acetic acid L-^−1^ in the fermentation broth.

Acetic acid, after pretreatment of unwashed samples, was generally <2% of the dry mass of corn stover. The maximum concentration was 1.1 g L^−1^ fermentation volume, which was observed for 75% moisture unensiled stover pretreated for 15 min. Only 8% of the unensiled samples, which had higher acetic acid concentrations than ensiled samples, had acetic acid concentrations exceeding 1 g L^−1^ (all at 65 and 75% moisture). Furfural and HMF concentrations in unwashed samples were 0.346 g L^−1^ ± 0.03 and 0.028 g L^−1^ ± 0.002, respectively. Although about 10% of the unwashed samples in this study had furfural levels exceeding 0.51 g L^−1^, the maximum HMF was below 0.06 g L^−1^. Maximum furfural concentration in unwashed samples was 0.73 g L^−1^ for 55% moisture ensiled stover pretreated for 15 min. The implication is that none of the potential inhibitors produced during pretreatment affected fermentation yield.

Even at higher solids loading, the concentrations in themselves may not contribute to significant inhibition. Graves et al. (2006) observed that with 25–30% solids in corn ethanol fermentation, *Saccharomyces cerevisiae* could tolerate, at pH ≥ 5, more than double the projected lactic acid concentration this study predicts would occur at 30% solids. There are several factors that complicate the predictability of the effects of these organic acids or furfural on ethanol yield under high solids loading. These include osmotic stress due to higher sugar concentrations (Darku and Richard, 2011); lower enzyme adsorption rates (Kristensen et al., 2009); mass transfer limitations (Varga et al., 2004; Kristensen et al., 2009; Zhang et al., 2009); and ethanol inhibition from higher ethanol concentration (Mohagheghi et al., 1992). Furthermore, these inhibitors act individually and synergetically, and their tolerable concentrations and combined effects are influenced by other factors, mainly pH (Palmqvist et al., 1999).

Disregarding these high solids limitations and scaling up the results from this study to reflect 30% solids loading, similar yields can be expected from both ensiled and unensiled stover based on the previously discussed observations by Palmqvist et al. (1999) and Larsson et al. (1999). This is because although ensiled samples have high furfural, only 14% of these samples at 30% solids loading would contain furfural >2.4 g L^−1^ (39% would be <1 g L^−1^) and maximum acetic acid would be <4 g L^−1^ (51% were ≤ 2 g L^−1^). At these concentrations, a stimulating effect on ethanol yield would be expected. For unensiled samples, acetic acid levels were higher than in ensiled samples. Yet even then, at a hypothetical 30% solids loading, while 89% of the samples would have concentrations >2 g L^−1^, only 4% would exceed 4.8 g L^−1^. In contrast, furfural concentrations were lower than in ensiled samples; at 30% solids the maximum concentration would be <2 g L^−1^ and 33% of the samples would have concentrations <1 g/L. With limited understanding of the complex interactions of inhibitors in fermentation broth coupled with the pH effect, it is possible that with higher solids, ensiled and unensiled feedstock could respond differently, with one having better outcomes over the other in terms of ethanol yield. While ethanol productivity will most certainly be affected by 15 min of pretreatment at higher solids loading, final yields may not be affected. Based on the current analysis, the ethanol fermentation outcomes of ensiled and unensiled samples at high solids would be balanced by lower acetic acid in the former and lower furfural in the later, likely resulting in similar yields.

Since wild type *Saccharomyces cerevisiae* is exclusively a 6-carbon fermenting yeast, the similarity in glucan composition of ensiled and unensiled samples also accounted for the similarity in ethanol yields. The assumption here is that there was no glucose degradation after structural decomposition during pretreatment. This assumption is based on the negligible amounts of HMF generated during pretreatment. At high temperature, under acidic conditions, glucans are degraded to HMF. In addition, washed samples did not have any HMF suggesting that HMF was likely from free water extractable glucose, which in the case of washed samples was washed out. The similarities in yields also suggest that although xylan removal was significantly higher in ensiled feedstock, it was not of practical significance for yeast hexose fermentation. Alternatively, it may be possible that glucan was more accessible in ensiled samples, but its utilization was hindered by inhibitors leading to coincidental similarities in yields with the unensiled samples. The following section, discussing fermentation yields with and without the pretreatment liquids, provides insight into this question.

#### Fermented With Pretreatment Extract vs. Without Extract

Ethanol yields for unwashed samples fermented with pretreatment extracts were significantly higher than samples fermented without extract (*p* = 0.041) (see Table 3), even after normalizing for the glucan removed during pretreatment that ended up in the extract. The amount of potential ethanol lost if extracts are discarded may seem negligible from a mass perspective. Glucan removal was low during pretreatment and on average could theoretically have yielded 0.0073 g ± 0.0002 of ethanol per sample, or 0.59 ± 0.04, g ethanol per liter fermentation volume. In terms of theoretical ethanol yield, this could potentially be up to 5% of the total ethanol that could be derived from the fermentation. Yields of samples fermented with and without extract were 50.57% ± 5.79 and 45.72% ± 6.01 g ethanol g^−1^ biomass, or on a mass ethanol per fermentation volume basis, 6.68 g L^−1^ ± 0.09 and 5.78 g L^−1^ ± 0.11, respectively.

The lower yields of samples without extract could be due to the absence of organic acids at the low, stimulating levels previously discussed. Correlation and regression analysis show that acetic acid has a positive correlation and significant regression relationship with ethanol yield in unensiled samples, but not with ensiled samples. This lack of correlation may be a result of lower acetic acid levels in the ensiled samples, suggesting that there may be a lower limit below which acetic acid has no stimulating effect, just as there is an upper limit as observed in other studies.

**Figure 3:**
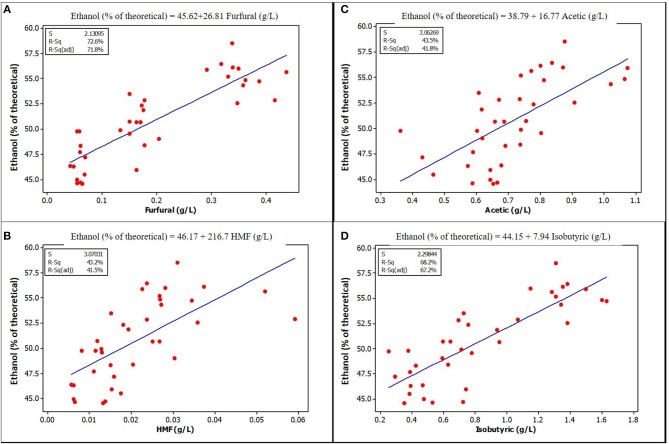
Relationships between ethanol yield and concentration of some potential inhibitors in the fermentation volume of unensiled stover that are significantly correlated **(A)** Furfural **(B)** HMF **(C)** Acetic acid **(D)** Isobutyric acid. See Supplementary Materials for correlation coefficients and *p*-values.

**Figure 4:**
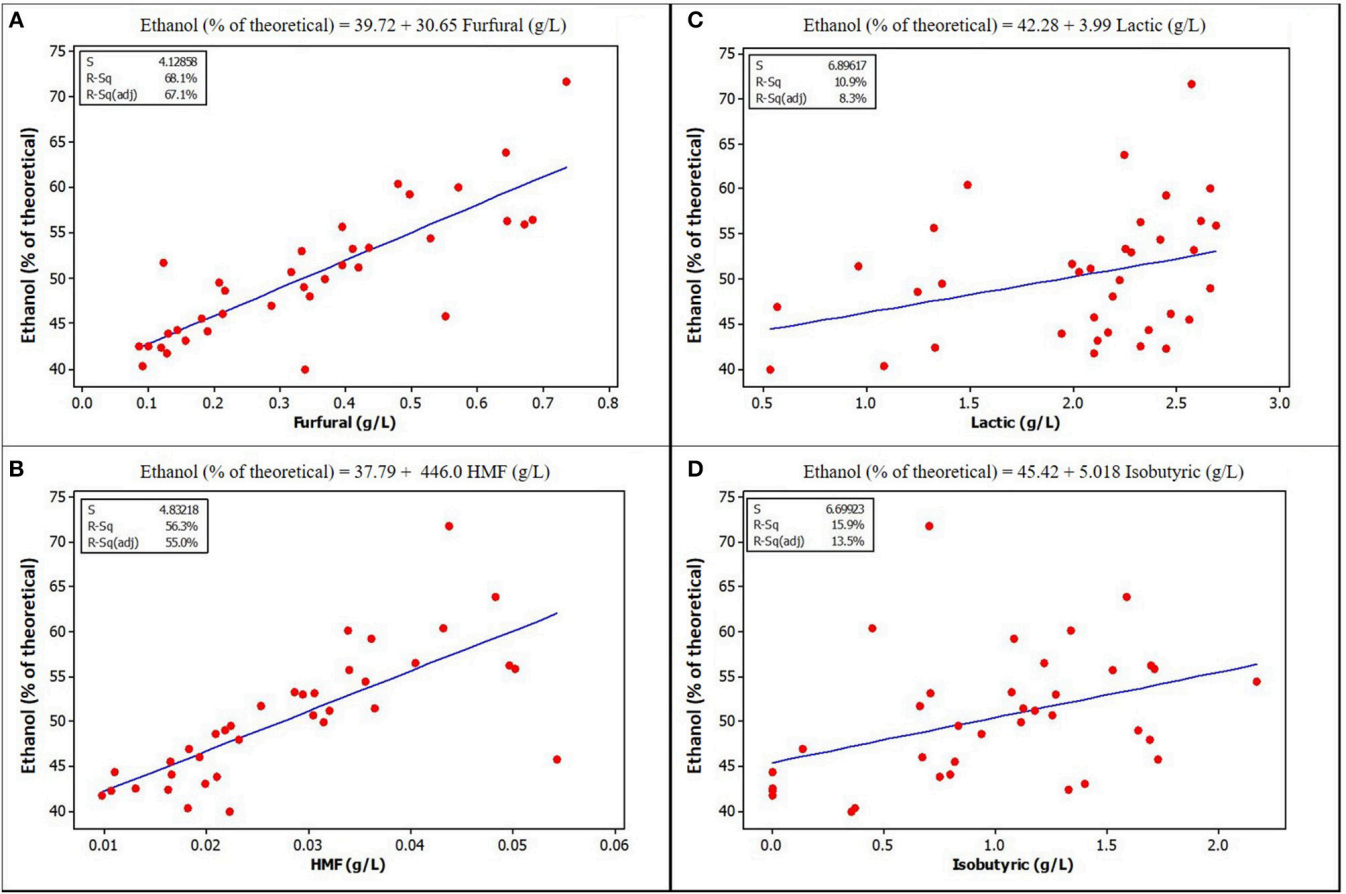
Relationships between ethanol yield and concentration of some potential inhibitors in the fermentation volume of ensiled stover. **(A)** Furfural **(B)** HMF [Lactic acid **(C)** and Isobutyric **(D)** are included because of their high concentration in ensiled samples, although they do not show obvious correlation with ethanol yield, *p*-values are also <0.05] See Supplementary Materials for correlation coefficients and *p*-values.

Furfural and HMF, at the concentrations found in unwashed stover in this study, were positively and strongly correlated with ethanol yield. Figures 3, 4 show a graphical relationship between some of these inhibitors and ethanol yield. The regression equations for unensiled stover were reasonable as they show that without furfural or HMF, ethanol yield was similar to the mean yield in samples fermented without pretreatment extract, hence with no furfural or HMF. This result supports the observation by Palmqvist et al. (1999) that furfural serves as an ethanol stimulant when concentrations are ≤ 2 g L^−1^. As noted in the previous paragraph, the organic acids, HMF and furfural in the pretreatment extract were not likely to exert any inhibition to fermentation and could be responsible for the higher yield in samples fermented with pretreatment extracts.

#### Storage at 23°C vs. Storage at 37°C

This section compares the ethanol yields of unwashed ensiled samples fermented with pretreatment extract at different storage temperatures. Samples stored at 23°C had better ethanol yields than samples stored at 37°C. These ethanol yields, reported as a percentage of theoretical ethanol yield, were 50.34% ± 7.20 at 23°C vs. 42.45% ± 7.53 at 37°C; *p* < 0.001). The difference, however, was due mainly to differences in yields at the extreme moisture levels, 25 and 75%. At these respective storage moistures of 25 and 75%, yields as percentage of theoretical were 47.99% ± 5.72 vs. 41.33% ± 3.76 and 49.12% ± 5.46 vs. 32.88% ± 6.56 for 23°C vs. 37°C. Although glucan composition before pretreatment and glucan removal during pretreatment were similar for samples ensiled at these two temperatures, xylan removal was significantly higher in samples stored at 23°C. In addition, samples stored at 23°C had higher acetic acid and lower lactic acid content in the pretreatment extracts than samples stored at 37°C. Thomas et al. (2002) observed that while both acetic acid and lactic acid at low concentrations could enhance ethanol yields, given appropriate pH, acetic acid was a better stimulant and resulted in more ethanol production while lactic acid benefited cell growth more than ethanol yield. At pH 4.5, increasing acetic acid concentration to 16 g L^−1^ did not have much inhibitory effect on yields (Thomas et al., 2002). Acetic acid levels in this study were lower and the pH of the fermentation media was approximately pH 4.8, so it is possible that this stimulating effect of acetic acid was responsible for the difference in yields.

#### Washed vs. Unwashed Samples

Samples stored at 37°C were analyzed for the effect of washing vs. not washing before pretreatment. Although unwashed samples fermented with the pretreatment extract had higher yields as a percentage of theoretical ethanol yields (42.45% ± 7.53) than washed samples (39.31% ± 6.55), there was no significant difference between the two. This was in spite of the higher xylan removed in the washed samples (36% DM) compared to unwashed samples (24% DM). The organic acid profiles of these two groups after pretreatment were also very different, with the washed samples dominated by malic acid, which is less inhibitory than the isobutyric, lactic and acetic acids that were the main acids in the unwashed samples. However, washed samples contained more than twice the amount of furfural found in unwashed samples.

On a furfural mass per fermentation volume basis, washed samples had 0.82 g L^−1^ ± 0.19 furfural while unwashed had 0.35 g L^−1^ ± 0.18 at a pretreatment retention time of 15 min. The furfural concentrations in the washed samples were not expected to inhibit ethanol production from discussion under fermentation of ensiled and unensiled stover. It is, however, possible that higher levels beyond 0.51 g L^−1^ (Xu et al., 2010) or 0.6 g L^−1^ (Palmqvist et al., 1999) could inhibit production in the presence of other compounds in the pretreatment extract. Boyer et al. (1992) found the growth rate of *S. cerevisiae* was not affected at furfural concentrations ≤ 1 g L^−1^ and specific ethanol productivity (gram ethanol per gram feedstock per hour, g/g/h) was not affected at 1.5 g L^−1^. Even at a furfural concentration of 2 g L^−1^, while specific ethanol productivity was significantly reduced, the final ethanol yield was not (Boyer et al., 1992). In the present study, fermentation was not allowed to proceed to completion, and that could have partially masked any inhibitory effect of borderline inhibitory concentrations of furfural, which are assumed to be responsible for the lower trending (but not significantly lower) yields of washed samples. The inhibitory effect of these compounds is dependent on several factors including pH, inoculation rate, substrate concentration, and the presence of other compounds.

Comparing washed samples fermented with extract to those fermented without extract, it was observed that although the ethanol yields of the latter were on average higher (45.65 % ± 6.62 vs. 41.56% ± 6.21, of theoretical ethanol, at 15 min pretreatment time), the difference was also not significant (*p* = 0.132). For the same pretreatment duration, and 37°C storage, ethanol yield of washed samples without extract were also not significantly different from that of unwashed samples fermented with extract, at 46.86% ± 11.29 of the theoretical ethanol yields. The implication is that although furfural may have some effect on fermentation at a concentration of 0.82 g L^−1^, this effect is not pronounced.

#### Effect of Storage Moisture and Pretreatment Retention Time

**Figure 5:**
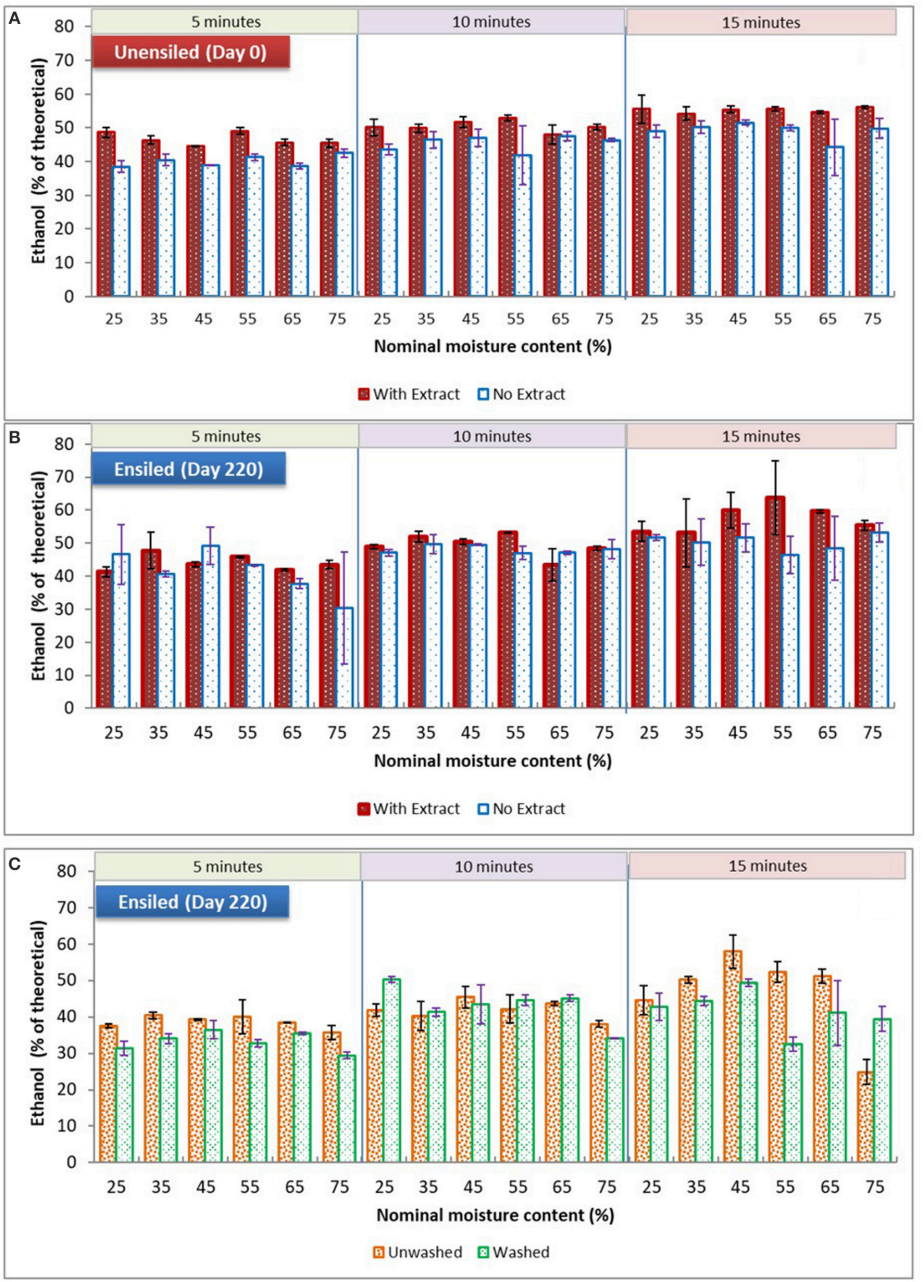
Comparing mean ethanol yields of dry ground stover at various moisture levels and pretreatment retention times. **(A)** and **(B)** are unwashed samples stored at 23°C ± 1, fermented with or without extracts; **(C)** is washed and unwashed samples stored at 37°C fermented with pretreatment extracts. Error bars are standard deviation of means.

With respect to the main processing alternatives investigated in this study, storage moisture had no significant influence on ethanol yield among all the samples of unwashed stover, both unensiled and ensiled at 23°C, whether fermented with or without their pretreatment extracts (see Figure 5). However, for unwashed samples ensiled at 37°C, there was a storage moisture effect. Although yields for 75% moisture samples [unwashed with extract] were not significantly different from 25% moisture, they were lower than yields from all other moisture treatments, averaging 37% of theoretical ethanol yield compared to yields >43% of theoretical for samples in the intermediate moisture range (*p* = 0.008). These high moisture samples were high in butyric and acetic acids and had no lactic acid prior to pretreatment. During pretreatment the butyric acid was eliminated, the acetic acid was reduced, and lactic acid was generated. The only marked differences between the 75% moisture samples and other samples were the lack of lactic acid and the high butyric acid concentrations. These differences are likely related, as lactic acid is known to be metabolized to butyric acid by clostridia during ensilage at high moisture levels (McDonald et al., 1991). This suggests that retaining high levels of lactic acid during storage, which is in a more dissociated form than the other organic acids due to storage pH, could have favorably impacted feedstock structure at other moisture levels. Although lactic acid at low concentrations can stimulate fermentation, these results as well as observations from previous paragraph suggest that maintaining lactic acid concentrations during storage is of greater value than generating lactic acid during pretreatment.

In contrast to storage moisture, pretreatment retention time frequently had a significant impact on ethanol yields. Generally, ethanol yields increased with pretreatment time. For unensiled samples, with and without their pretreatment extracts, yields at the various pretreatment times were significantly different from each other. Importantly, for ensiled samples, yields from the 10-min pretreatment duration was not significantly different from 15 min, except for the 23°C unwashed samples fermented with extract. This suggests that ensilage may permit a reduction of pretreatment severity without sacrificing yield, potentially reducing conversion costs. These pretreatment time comparisons are detailed in Table 3. (For data used in used in analysis made in this study, section Results and discussion, see the Supplementary Material).

## Conclusions

The results of this study indicate that the organic acids produced during wet storage and/or pretreatment generally do not inhibit ethanol fermentation and instead can enhance the fermentation yield. These effects of organic acids can be observed at three levels: (1) at the storage level, they potentially alter feedstock structure, resulting in more xylan removal, or weaker linkages between components of the plant cell wall matrix, (2) during subsequent pretreatment, when organic acids can accelerate as well as limit xylan removal depending on the acids involved, and (3) during fermentation, when organic acids, and some other pretreatment products known be inhibitory to yeast at high concentrations can instead provide a minor benefit at low concentrations. The contributions of organic acids to the downstream conversion of corn stover feedstock to ethanol are greater at the storage level, and the partial pretreatment benefits are more pronounced during storage than in subsequent thermochemical pretreatment processing. The organic acids produced during this wet storage study were not inhibitory to *S. cerevisiae*, and interactions of the acids with conversion processes leading to the final ethanol products were mostly positive.

Lactic acid, which is less inhibitory than some other organic acids, was dominant in ensiled samples and its low pKa means more of it was in the dissociated form. Higher levels of dissociated acids mean more hydrogen ions that can interact favorably with structural bonds. When these effects occur at the storage level the disassociated acids cannot be easily assimilated into microbes even if retained in subsequent processes and are thus less likely to inhibit microbial growth or ethanol production.

An important observation of this study is that the acid profile that was generated during wet storage changed during pretreatment. When lactic acid is dominant during storage there is the potential for production of more acetic acid during LHW pretreatment. This sequence benefits the downstream fermentation, since acetic acid is a better ethanol stimulant than lactic acid. As has long been observed in the livestock forage industry (McDonald et al., 1991), lactic acid dominated silage has the best outcomes during both storage and in subsequent bioconversion. From an engineering design perspective, it would be useful to develop coupled ensilage/pretreatment systems that encourage more lactic acid production during storage and more acetic acid in subsequent pretreatment processing, as long as those acid concentrations are <5% DM.

Using both xylan removal and ethanol yield as proxies for pretreatment effectiveness, these results also provide evidence that pretreatment of ensiled stover could be carried out at shorter pretreatment times and thus lower severity than unensiled stover, and still be as effective. There was evidence from the xylan removal results that wet storage resulted in changes that rendered the feedstock more responsive to subsequent pretreatment process. Fermentation results also indicated ensiled stover could achieve similar ethanol yields with shortened pretreatment times. However, as pretreatment severity increases, the benefits derived from ensilage decrease. Xylan removal rates by themselves were not always predictive, providing an indication of pretreatment severity, and perhaps pretreatment effectiveness, but not necessarily fermentation outcomes, perhaps due to the presence of other compounds generated during storage or pretreatment.

In general, if extreme storage moistures (25 and 75%) are avoided, the impacts of moisture on subsequent process outcomes, especially respect to ethanol yields, are not significant. This is also good news for biorefineries, which thus do not have to be overly concerned with process adjustments to accommodate changes that might result from different storage moistures, which are difficult to control in an industrial feedstock supply chain.

This study documents multiple benefits and few limitations associated with wet storage of biomass for ethanol fermentation. As the feedstock supply chains for lignocellulosic biofuels expand to millions and eventually billions of tons worldwide, wet storage can be an effective strategy for both preserving biomass and enhancing downstream conversion processes.

## Author Contributions

DE and TR conceived the idea. DE designed, planned and performed the experiments. TR provided valuable feedback that shaped the research as it progressed. DE performed the data analysis, discussed results with TR, drafted the manuscript and designed the figures. TR reviewed and edited the manuscript.

### Conflict of Interest Statement

The authors declare that the research was conducted in the absence of any commercial or financial relationships that could be construed as a potential conflict of interest.
