# Supplementary material for: Ensiled Wet Storage Accelerates Pretreatment for Bioconversion of Corn Stover
Source: Front Bioeng Biotechnol. 2018 Dec 14;6:195. doi: 10.3389/fbioe.2018.00195 (PMC6302026; doi:10.3389/fbioe.2018.00195)
Supplement: Supplementary file 1 [file Data_Sheet_1.docx]

SUPPLEMENTARY MATERIALS

Data in this supplementary section are mean values along with the standard deviation of means as a measure of dispersion. For each moisture adjusted sample ensiled, a corresponding sample of the same moisture was kept aside as an unensiled (Day 0) control. These unensiled samples were analyzed within 24 hours for pH, organic acids and the composition analysis was carried out over 6 days. The unensiled samples were dried in a HotPack convection oven at 55^o^C, ground using a 2 mm screen on a Wiley Mill (Model 4, Thomas Scientific, Swedesboro, NJ) and stored at room temperature in sterile airtight Whirl-Pak bags (Nasco, Fort Atkinson, Wisc.) to be pretreated and fermented alongside the ensiled samples. Temperature values for Day 0 are just for the purpose of associating the Day 0 samples, which were used as controls, with their corresponding ensiled, Day 220 samples.


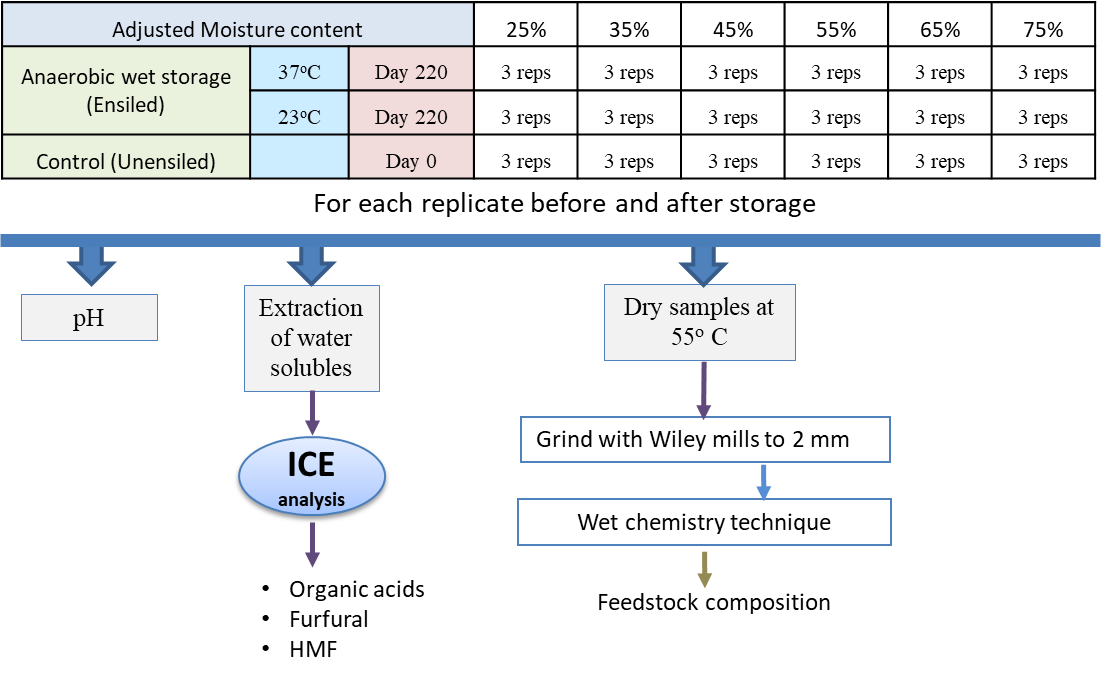


Figure S1: Flowchart for storage experiment. Samples were extracted in water at room temperature and a stover water ratio of 1:10. The mixtures were shaken for 30 minutes at 200 rpm using a Barnstead SHKA 2000 open air platform shaker (Barnstead International, Dubuque, IA). Extracts were filtered and analyzed for listed compounds using pH meter (SevenEasy S20, Mettler-Toledo International Inc, Columbus, OH) and Dionex ion chromatograph (ICS 3000, Thermo Fisher Scientific Inc., Sunnyvale, CA). Feedstock composition, including structural carbohydrates and lignin, were analyzed using NREL standard protocols (Hames et al., 2008; Sluiter et al., 2008a, 2008b, 2008c)


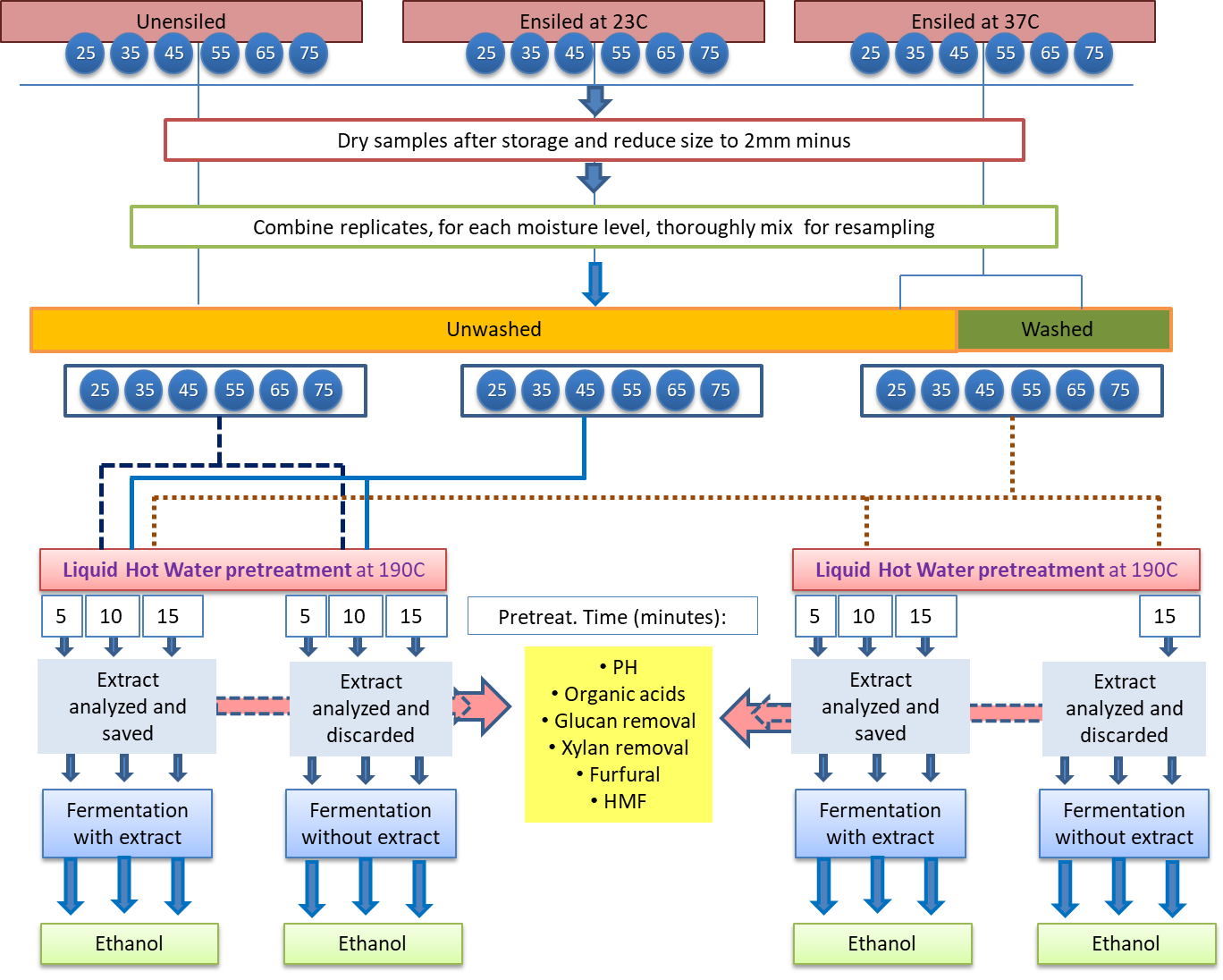


Figure S2: Process flow chart for pretreatment and ethanol fermentation. For each moisture level (25% to 75%, wet basis), there were two replicates at each pretreatment time. Only 37^o^C samples were washed for comparison with unwashed samples.

Table S1: Organic acids composition before pretreatment*

| Storage Duration | Storage temperature (^o^C) | Nominal moisture content (%) | Lactic Acid (% DM) | Acetic Acid (% DM) | Propionic Acid (% DM) | Isobutyric Acid (% DM) | Butyric Acid (% DM) |
| --- | --- | --- | --- | --- | --- | --- | --- |
| Day 0  (unensiled) | 23 | 25 | 0.097 ± 0.096 | 0.000 ± 0.000 | 0.000 ± 0.000 | 0.03 ± 0.052 | 0.000 ± 0.000 |
|  | 23 | 35 | 0.357 ± 0.309 | 0.000 ± 0.000 | 0.000 ± 0.000 | 0.05 ± 0.044 | 0.000 ± 0.000 |
|  | 23 | 45 | 0.185 ± 0.008 | 0.000 ± 0.000 | 0.000 ± 0.000 | 0.085 ± 0.008 | 0.000 ± 0.000 |
|  | 23 | 55 | 0.130 ± 0.226 | 0.000 ± 0.000 | 0.000 ± 0.000 | 0.034 ± 0.058 | 0.000 ± 0.000 |
|  | 23 | 65 | 0.000 ± 0.000 | 0.000 ± 0.000 | 0.000 ± 0.000 | 0.000 ± 0.000 | 0.000 ± 0.000 |
|  | 23 | 75 | 0.000 ± 0.000 | 0.000 ± 0.000 | 0.000 ± 0.000 | 0.000 ± 0.000 | 0.000 ± 0.000 |
|  | 37 | 25 | 0.000 ± 0.000 | 0.000 ± 0.000 | 0.000 ± 0.000 | 0.000 ± 0.000 | 0.000 ± 0.000 |
|  | 37 | 35 | 0.000 ± 0.000 | 0.000 ± 0.000 | 0.000 ± 0.000 | 0.000 ± 0.000 | 0.000 ± 0.000 |
|  | 37 | 45 | 0.000 ± 0.000 | 0.054 ± 0.093 | 0.000 ± 0.000 | 0.000 ± 0.000 | 0.000 ± 0.000 |
|  | 37 | 55 | 0.000 ± 0.000 | 0.000 ± 0.000 | 0.000 ± 0.000 | 0.000 ± 0.000 | 0.000 ± 0.000 |
|  | 37 | 65 | 0.000 ± 0.000 | 0.000 ± 0.000 | 0.000 ± 0.000 | 0.000 ± 0.000 | 0.000 ± 0.000 |
|  | 37 | 75 | 0.000 ± 0.000 | 0.000 ± 0.000 | 0.000 ± 0.000 | 0.000 ± 0.000 | 0.000 ± 0.000 |
| Day 220  (ensiled) | 23 | 25 | 0.800 ± 0.270 | 0.370 ± 0.066 | 0.000 ± 0.000 | 0.054 ± 0.093 | 0.000 ± 0.000 |
|  | 23 | 35 | 2.520 ± 0.380 | 0.907 ± 0.370 | 0.000 ± 0.000 | 0.107 ± 0.006 | 0.000 ± 0.000 |
|  | 23 | 45 | 2.787 ± 0.444 | 0.860 ± 0.152 | 0.000 ± 0.000 | 0.107 ± 0.006 | 0.000 ± 0.000 |
|  | 23 | 55 | 3.200 ± 0.471 | 1.350 ± 0.424 | 0.000 ± 0.000 | 0.080 ± 0.070 | 0.000 ± 0.000 |
|  | 23 | 65 | 2.900 ± 0.572 | 1.017 ± 0.886 | 0.000 ± 0.000 | 0.730 ± 0.102 | 0.000 ± 0.000 |
|  | 23 | 75 | 2.900 ± 0.798 | 1.857 ± 0.203 | 0.104 ± 0.179 | 1.194 ± 0.065 | 0.000 ± 0.000 |
|  | 37 | 25 | 0.264 ± 0.457 | 0.137 ± 0.237 | 0.000 ± 0.000 | 0.040 ± 0.070 | 0.000 ± 0.000 |
|  | 37 | 35 | 1.944 ± 0.126 | 0.627 ± 0.016 | 0.000 ± 0.000 | 0.200 ± 0.018 | 0.000 ± 0.000 |
|  | 37 | 45 | 2.850 ± 0.198 | 0.735 ± 0.078 | 0.000 ± 0.000 | 0.270 ± 0.015 | 0.000 ± 0.000 |
|  | 37 | 55 | 2.954 ± 0.396 | 1.144 ± 0.191 | 0.000 ± 0.000 | 0.337 ± 0.220 | 0.000 ± 0.000 |
|  | 37 | 65 | 3.034 ± 1.760 | 1.237 ± 0.206 | 0.000 ± 0.000 | 0.157 ± 0.272 | 0.000 ± 0.000 |
|  | 37 | 75 | 0.000 ± 0.000 | 2.744 ± 0.726 | 0.000 ± 0.000 | 1.280 ± 1.063 | 2.570 ± 0.581 |

Table S2: Sugar removal and inhibitors generated during pretreatment of unwashed and washed 37^o^C samples†

| Storage Duration | Pretreatment retention time (min) | Nominal storage moisture (%) | Glucan removed (%) | Xylan removed (%) | Furfural generated (%) | HMF generated (%) |
| --- | --- | --- | --- | --- | --- | --- |
| Day 220 | 5 | 25 | 3.76 ± 0.50 | 23.94 ± 2.74 | 0.31 ± 0.31 | 0.03 ± 0.03 |
|  | 5 | 35 | 3.08 ± 0.61 | 22.75 ± 2.64 | 0.16 ± 0.04 | 0.03 ± 0.01 |
|  | 5 | 45 | 3.18 ± 0.20 | 30.15 ± 2.86 | 0.32 ± 0.13 | 0.05 ± 0.02 |
|  | 5 | 55 | 3.02 ± 0.07 | 27.63 ± 0.72 | 0.22 ± 0.12 | 0.03 ± 0.01 |
|  | 5 | 65 | 2.68 ± 0.14 | 24.37 ± 1.19 | 0.12 ± 0.01 | 0.01 ± 0.00 |
|  | 5 | 75 | 2.07 ± 0.21 | 18.86 ± 1.25 | 0.19 ± 0.04 | 0.02 ± 0.01 |
|  | 10 | 25 | 3.71 ± 0.04 | 27.14 ± 0.33 | 0.33 ± 0.04 | 0.03 ± 0.00 |
|  | 10 | 35 | 2.94 ± 0.20 | 23.36 ± 0.95 | 0.41 ± 0.01 | 0.05 ± 0.01 |
|  | 10 | 45 | 2.88 ± 0.15 | 29.03 ± 1.42 | 0.54 ± 0.16 | 0.06 ± 0.01 |
|  | 10 | 55 | 2.96 ± 0.01 | 28.09 ± 0.83 | 0.38 ± 0.21 | 0.03 ± 0.01 |
|  | 10 | 65 | 2.88 ± 0.33 | 25.24 ± 1.85 | 0.33 ± 0.01 | 0.02 ± 0.00 |
|  | 10 | 75 | 2.81 ± 0.20 | 27.01 ± 1.39 | 0.41 ± 0.11 | 0.03 ± 0.01 |
|  | 15 | 25 | 2.74 ± 1.23 | 21.93 ± 5.78 | 0.60 ± 0.12 | 0.05 ± 0.01 |
|  | 15 | 35 | 2.80 ± 0.04 | 22.74 ± 1.54 | 0.35 ± 0.40 | 0.04 ± 0.03 |
|  | 15 | 45 | 2.89 ± 0.04 | 24.81 ± 1.57 | 0.78 ± 0.01 | 0.06 ± 0.00 |
|  | 15 | 55 | 3.18 ± 0.16 | 26.96 ± 1.01 | 0.54 ± 0.44 | 0.05 ± 0.04 |
|  | 15 | 65 | 2.74 ± 0.01 | 23.62 ± 2.34 | 0.30 ± 0.23 | 0.02 ± 0.01 |
|  | 15 | 75 | 2.53 ± 0.18 | 23.28 ± 2.35 | 0.50 ± 0.20 | 0.04 ± 0.01 |
|  | WASHED |  |  |  |  |  |
|  | 15 | 25 | 3.43 ± 0.55 | 31.75 ± 1.95 | 0.93 ± 0.05 | ND* |
|  | 15 | 35 | 3.41 ± 0.52 | 38.62 ± 6.75 | 1.20 ± 0.10 | ND |
|  | 15 | 45 | 3.32 ± 0.34 | 39.60 ± 5.13 | 1.33 ± 0.06 | ND |
|  | 15 | 55 | 3.24 ± 0.37 | 36.97 ± 2.72 | 1.41 ± 0.02 | ND |
|  | 15 | 65 | 2.67 ± 0.14 | 32.77 ± 0.88 | 1.19 ± 0.06 | ND |
|  | 15 | 75 | 2.74 ± 0.41 | 32.85 ± 1.53 | 0.69 ± 0.01 | ND |

† Washed data are for samples fermented without extract. Glucan and xylan removal are percentage of the total glucan (or xylan) polymer in the feedstock before pretreatment. Furfural and HMF generated are percentage of dry matter. Same definitions apply to Table S3.

*ND = Not detected

Table S3: Sugar removal and inhibitors generated during pretreatment of unwashed 23^o^C samples (includes replicates of samples fermented with and without pretreatment extract)

| Storage Duration | Pretreatment retention time (min) | Nominal storage moisture (%) | Glucan removed (%) | Xylan removed (%) | Furfural generated (%) | HMF generated (%) |
| --- | --- | --- | --- | --- | --- | --- |
| Day 220 | 5 | 25 | 3.35 ± 0.18 | 25.28 ± 0.18 | 0.22 ± 0.21 | 0.03 ± 0.01 |
|  | 5 | 35 | 3.34 ± 0.83 | 29.52 ± 2.53 | 0.19 ± 0.01 | 0.03 ± 0.01 |
|  | 5 | 45 | 2.64 ± 0.45 | 24.70 ± 3.19 | 0.29 ± 0.08 | 0.04 ± 0.02 |
|  | 5 | 55 | 3.74 ± 1.35 | 23.91 ± 0.03 | 0.24 ± 0.07 | 0.02 ± 0.01 |
|  | 5 | 65 | 3.16 ± 0.99 | 27.34 ± 0.90 | 0.16 ± 0.03 | 0.01 ± 0.01 |
|  | 5 | 75 | 3.8 ± 1.69 | 29.10 ± 0.75 | 0.20 ± 0.03 | 0.02 ± 0.01 |
|  | 10 | 25 | 3.12 ± 0.26 | 26.88 ± 3.01 | 0.32 ± 0.04 | 0.03 ± 0.00 |
|  | 10 | 35 | 3.00 ± 0.39 | 30.42 ± 1.35 | 0.47 ± 0.04 | 0.05 ± 0.01 |
|  | 10 | 45 | 2.43 ± 0.35 | 30.09 ± 1.62 | 0.52 ± 0.06 | 0.05 ± 0.01 |
|  | 10 | 55 | 3.65 ± 1.26 | 32.16 ± 0.30 | 0.48 ± 0.18 | 0.04 ± 0.01 |
|  | 10 | 65 | 3.50 ± 1.47 | 27.70 ± 3.86 | 0.45 ± 0.03 | 0.03 ± 0.00 |
|  | 10 | 75 | 3.19 ± 0.67 | 27.68 ± 1.44 | 0.47 ± 0.04 | 0.03 ± 0.00 |
|  | 15 | 25 | 3.46 ± 0.22 | 26.24 ± 0.53 | 0.59 ± 0.08 | 0.05 ± 0.01 |
|  | 15 | 35 | 2.92 ± 0.85 | 27.51 ± 1.87 | 0.59 ± 0.34 | 0.06 ± 0.02 |
|  | 15 | 45 | 2.58 ± 0.25 | 24.28 ± 0.29 | 0.94 ± 0.02 | 0.07 ± 0.01 |
|  | 15 | 55 | 3.64 ± 1.00 | 27.17 ± 1.32 | 0.79 ± 0.37 | 0.06 ± 0.02 |
|  | 15 | 65 | 2.79 ± 2.00 | 19.88 ± 5.17 | 0.68 ± 0.13 | 0.05 ± 0.01 |
|  | 15 | 75 | 2.98 ± 0.38 | 30.44 ± 1.64 | 0.89 ± 0.12 | 0.05 ± 0.01 |
| Day 0 | 5 | 25 | 3.74 ± 0.82 | 22.27 ± 1.28 | 0.07 ± 0.04 | 0.02 ± 0.01 |
|  | 5 | 35 | 3.29 ± 1.31 | 23.52 ± 0.84 | 0.09 ± 0.01 | 0.02 ± 0.01 |
|  | 5 | 45 | 3.61 ± 1.25 | 21.65 ± 0.05 | 0.09 ± 0.00 | 0.02 ± 0.00 |
|  | 5 | 55 | 4.26 ± 0.45 | 22.84 ± 2.82 | 0.08 ± 0.01 | 0.02 ± 0.01 |
|  | 5 | 65 | 3.66 ± 0.83 | 19.10 ± 0.72 | 0.08 ± 0.01 | 0.01 ± 0.00 |
|  | 5 | 75 | 5.32 ± 0.34 | 22.30 ± 4.50 | 0.07 ± 0.01 | 0.01 ± 0.00 |
|  | 10 | 25 | 3.94 ± 0.66 | 28.76 ± 0.21 | 0.26 ± 0.01 | 0.03 ± 0.01 |
|  | 10 | 35 | 3.76 ± 1.27 | 28.64 ± 1.60 | 0.25 ± 0.03 | 0.04 ± 0.01 |
|  | 10 | 45 | 3.12 ± 2.44 | 26.79 ± 3.25 | 0.25 ± 0.01 | 0.03 ± 0.01 |
|  | 10 | 55 | 4.31 ± 0.74 | 29.46 ± 0.24 | 0.23 ± 0.03 | 0.03 ± 0.01 |
|  | 10 | 65 | 4.41 ± 0.63 | 23.76 ± 3.15 | 0.22 ± 0.02 | 0.02 ± 0.00 |
|  | 10 | 75 | 4.84 ± 1.06 | 25.99 ± 1.65 | 0.21 ± 0.02 | 0.02 ± 0.00 |
|  | 15 | 25 | 3.80 ± 0.80 | 27.70 ± 1.30 | 0.49 ± 0.02 | 0.05 ± 0.00 |
|  | 15 | 35 | 3.96 ± 1.33 | 27.25 ± 0.64 | 0.55 ± 0.08 | 0.07 ± 0.02 |
|  | 15 | 45 | 3.66 ± 1.44 | 26.88 ± 1.81 | 0.53 ± 0.03 | 0.05 ± 0.01 |
|  | 15 | 55 | 4.35 ± 0.78 | 27.71 ± 1.82 | 0.47 ± 0.05 | 0.04 ± 0.00 |
|  | 15 | 65 | 4.03 ± 0.60 | 26.71 ± 0.64 | 0.49 ± 0.05 | 0.04 ± 0.00 |
|  | 15 | 75 | 5.00 ± 0.29 | 26.34 ± 2.35 | 0.41 ± 0.04 | 0.03 ± 0.00 |

Table S4: Composition (% dry matter) of IA stover before and after anaerobic storage at 23^o^C (black) & 37^o^C (red)

- Significant components of water-soluble extractives include water soluble carbohydrates (monosaccharides― e.g. glucose, xylose, sucrose ― and oligomeric sugars); organic acids; alditols; inorganic ions/cations; tannins
- Ethanol extractives could include waxes, fats, resins, gums, phytosterols, chlorophyll
- Nonstructural inorganics could include dust or soil contaminants or other components introduced during biomass collection or handling
- Structural inorganics are mainly ash, which could contain any variety of mineral matter composition; silicates, metal oxides and hydroxides; sulphates, sulphides, sulphosalts; carbonates and bicarbonates; elemental minerals, etc.

Table S5: Change in hemicellulose and acetyl content after storage*+

*Change in hemicellulose and acetyl reflect amount degraded during storage and calculated as:

$$\frac{\left( g Hemicellulose or acetyl,Day 0-g Hemicellulose or acetyl,Day 220 \right)}{g Hemicellulose or acetyl,Day 0}\times100$$

Table S6: Organic acids and pH after pretreatment of unwashed 23^o^C samples

| Storage duration | Pretreatment retention time (min) | Nominal storage moisture (%) | pH | Acetic acid (% DM) | Lactic acid (% DM) | Isobutyric acid (% DM) | Formic acid (% DM) | Malic acid (% DM) | Pyruvic acid (% DM) | Tartaric acid (% DM) | Total acids (% DM) |
| --- | --- | --- | --- | --- | --- | --- | --- | --- | --- | --- | --- |
| Day 220 | 5 | 25 | 4.52 ± 0.19 | 0.69 ± 0.18 | 1.68 ± 0.15 | 1.15 ± 0.57 | 0.2 ± 0.08 | 0.12 ± 0.09 | 0.22 ± 0.11 | 0.72 ± 0.69 | 4.78 ± 0.84 |
|  | 5 | 35 | 4.44 ± 0.07 | 0.53 ± 0.14 | 2.38 ± 0.58 | 1.1 ± 0.37 | 0.17 ± 0.06 | 0.23 ± 0.06 | 0.24 ± 0.06 | 0.31 ± 0.09 | 4.94 ± 0.80 |
|  | 5 | 45 | 4.24 ± 0.07 | 0.55 ± 0.23 | 3.15 ± 0.26 | 1.46 ± 0.41 | 0.18 ± 0.06 | 0.93 ± 1.66 | 0.26 ± 0.14 | 0.26 ± 0.17 | 6.78 ± 2.30 |
|  | 5 | 55 | 4.21 ± 0.09 | 0.35 ± 0.25 | 3.60 ± 0.17 | 1.18 ± 0.15 | 0.15 ± 0.02 | 0.67 ± 1.11 | 0.23 ± 0.14 | 0.18 ± 0.16 | 6.36 ± 0.74 |
|  | 5 | 65 | 4.43 ± 0.06 | 0.48 ± 0.02 | 3.41 ± 0.24 | 0.00 ± 0.00 | 0.13 ± 0.01 | 0.05 ± 0.01 | 0.14 ± 0.00 | 0.01 ± 0.01 | 4.20 ± 0.23 |
|  | 5 | 75 | 4.37 ± 0.03 | 0.53 ± 0.05 | 3.46 ± 0.05 | 0.00 ± 0.00 | 0.14 ± 0.01 | 0.08 ± 0.01 | 0.14 ± 0.01 | 0.03 ± 0.01 | 4.40 ± 0.03 |
|  | 10 | 25 | 4.36 ± 0.05 | 0.55 ± 0.36 | 1.76 ± 0.19 | 1.30 ± 0.06 | 0.21 ± 0.01 | 0.14 ± 0.02 | 0.21 ± 0.05 | 0.39 ± 0.26 | 4.55 ± 0.19 |
|  | 10 | 35 | 4.28 ± 0.05 | 0.79 ± 0.07 | 2.87 ± 0.36 | 1.77 ± 0.09 | 0.26 ± 0.03 | 0.19 ± 0.04 | 0.23 ± 0.05 | 0.35 ± 0.10 | 6.46 ± 0.49 |
|  | 10 | 45 | 4.15 ± 0.05 | 0.46 ± 0.54 | 3.12 ± 0.09 | 2.08 ± 0.51 | 0.17 ± 0.11 | 1.16 ± 1.53 | 0.28 ± 0.15 | 0.09 ± 0.11 | 7.36 ± 2.12 |
|  | 10 | 55 | 4.15 ± 0.12 | 0.39 ± 0.39 | 3.52 ± 0.21 | 1.59 ± 0.41 | 0.19 ± 0.05 | 0.78 ± 0.81 | 0.40 ± 0.17 | 0.00 ± 0.00 | 6.88 ± 1.18 |
|  | 10 | 65 | 4.26 ± 0.07 | 0.46 ± 0.33 | 2.14 ± 1.54 | 1.20 ± 0.99 | 1.1 ± 0.99 | 1.86 ± 1.92 | 0.10 ± 0.11 | 0.8 ± 1.45 | 7.66 ± 1.44 |
|  | 10 | 75 | 4.19 ± 0.10 | 0.86 ± 0.03 | 3.46 ± 0.30 | 2.52 ± 0.19 | 0.29 ± 0.05 | 0.31 ± 0.21 | 0.16 ± 0.01 | 0.03 ± 0.01 | 7.63 ± 0.45 |
|  | 15 | 25 | 4.35 ± 0.17 | 0.87 ± 0.06 | 1.71 ± 0.25 | 1.83 ± 0.35 | 0.27 ± 0.05 | 0.23 ± 0.03 | 0.23 ± 0.04 | 0.34 ± 0.18 | 5.47 ± 0.47 |
|  | 15 | 35 | 4.21 ± 0.21 | 0.88 ± 0.61 | 2.50 ± 0.40 | 2.30 ± 1.13 | 0.34 ± 0.13 | 0.36 ± 0.25 | 0.25 ± 0.03 | 0.20 ± 0.10 | 6.83 ± 2.40 |
|  | 15 | 45 | 4.11 ± 0.07 | 0.62 ± 0.31 | 3.29 ± 0.07 | 2.29 ± 0.30 | 0.29 ± 0.04 | 0.58 ± 0.48 | 0.2 ± 0.020 | 0.35 ± 0.15 | 7.61 ± 0.96 |
|  | 15 | 55 | 4.11 ± 0.16 | 0.50 ± 0.46 | 3.79 ± 0.11 | 2.20 ± 0.79 | 0.26 ± 0.09 | 1.68 ± 1.51 | 0.28 ± 0.17 | 0.05 ± 0.10 | 8.76 ± 2.19 |
|  | 15 | 65 | 4.14 ± 0.15 | 1.00 ± 0.11 | 3.59 ± 0.23 | 2.26 ± 0.61 | 0.36 ± 0.06 | 0.67 ± 0.32 | 0.20 ± 0.02 | 0.01 ± 0.01 | 8.08 ± 0.86 |
|  | 15 | 75 | 4.18 ± 0.18 | 1.04 ± 0.08 | 3.61 ± 0.20 | 2.93 ± 0.79 | 0.36 ± 0.03 | 1.00 ± 0.52 | 0.19 ± 0.01 | 0.00 ± 0.00 | 9.15 ± 0.92 |

Table S6 cont:

| Storage duration | Pretreatment retention time (min) | Nominal storage moisture (%) | pH | Acetic acid (% DM) | Lactic acid (% DM) | Isobutyric acid (% DM) | Formic acid (% DM) | Malic acid (% DM) | Pyruvic acid (% DM) | Tartaric acid (% DM) | Total acids (% DM) |
| --- | --- | --- | --- | --- | --- | --- | --- | --- | --- | --- | --- |
| Day 0 | 5 | 25 | 4.64 ± 0.06 | 0.76 ± 0.22 | 0.00 ± 0.00 | 0.61 ± 0.19 | 0.13 ± 0.08 | 0.12 ± 0.06 | 0.20 ± 0.28 | 0.58 ± 0.96 | 2.40 ± 1.61 |
|  | 5 | 35 | 4.61 ± 0.09 | 0.83 ± 0.21 | 0.00 ± 0.00 | 0.49 ± 0.07 | 0.16 ± 0.03 | 0.13 ± 0.04 | 0.48 ± 0.03 | 0.83 ± 0.12 | 2.91 ± 0.37 |
|  | 5 | 45 | 4.62 ± 0.09 | 0.86 ± 0.14 | 0.35 ± 0.49 | 0.77 ± 0.34 | 0.17 ± 0.02 | 0.19 ± 0.02 | 0.35 ± 0.13 | 0.75 ± 0.88 | 3.43 ± 1.12 |
|  | 5 | 55 | 4.64 ± 0.08 | 0.91 ± 0.12 | 0.27 ± 0.35 | 0.74 ± 0.17 | 0.15 ± 0.02 | 0.1 ± 0.03 | 0.36 ± 0.10 | 1.08 ± 0.31 | 3.60 ± 0.71 |
|  | 5 | 65 | 4.61 ± 0.15 | 0.79 ± 0.39 | 0.00 ± 0.00 | 0.74 ± 0.12 | 0.12 ± 0.08 | 0.07 ± 0.02 | 1.46 ± 0.80 | 2.76 ± 0.73 | 5.93 ± 1.37 |
|  | 5 | 75 | 4.71 ± 0.03 | 1.21 ± 0.33 | 0.00 ± 0.01 | 0.82 ± 0.11 | 0.32 ± 0.33 | 0.07 ± 0.03 | 0.79 ± 0.43 | 1.67 ± 1.84 | 4.88 ± 2.78 |
|  | 10 | 25 | 4.43 ± 0.07 | 0.99 ± 0.13 | 0.00 ± 0.02 | 1.18 ± 0.23 | 0.26 ± 0.02 | 0.22 ± 0.03 | 0.50 ± 0.22 | 0.33 ± 0.43 | 3.47 ± 0.53 |
|  | 10 | 35 | 4.37 ± 0.07 | 0.95 ± 0.05 | 0.28 ± 0.55 | 1.24 ± 0.4 | 0.23 ± 0.04 | 0.19 ± 0.07 | 0.43 ± 0.11 | 0.75 ± 0.26 | 4.07 ± 0.85 |
|  | 10 | 45 | 4.39 ± 0.08 | 1.02 ± 0.17 | 0.21 ± 0.33 | 1.28 ± 0.41 | 0.27 ± 0.03 | 0.31 ± 0.15 | 0.31 ± 0.07 | 0.06 ± 0.07 | 3.44 ± 0.49 |
|  | 10 | 55 | 4.44 ± 0.07 | 0.98 ± 0.12 | 0.34 ± 0.40 | 1.31 ± 0.25 | 0.24 ± 0.03 | 0.2 ± 0.08 | 0.35 ± 0.09 | 0.16 ± 0.23 | 3.57 ± 0.36 |
|  | 10 | 65 | 4.4 ± 0.12 | 0.82 ± 0.37 | 0.00 ± 0.00 | 1.88 ± 1.35 | 0.83 ± 0.91 | 0.94 ± 1.56 | 1.49 ± 1.04 | 0.88 ± 1.59 | 6.84 ± 4.42 |
|  | 10 | 75 | 4.5 ± 0.06 | 1.41 ± 0.50 | 0.00 ± 0.01 | 1.32 ± 0.39 | 0.55 ± 0.58 | 0.82 ± 1.4 | 0.49 ± 0.59 | 0.18 ± 0.22 | 4.76 ± 3.20 |
|  | 15 | 25 | 4.26 ± 0.05 | 1.22 ± 0.20 | 0.00 ± 0.02 | 2.06 ± 0.15 | 0.28 ± 0.13 | 0.28 ± 0.04 | 0.37 ± 0.05 | 0.56 ± 0.47 | 4.76 ± 0.32 |
|  | 15 | 35 | 4.26 ± 0.09 | 1.19 ± 0.15 | 0.56 ± 0.65 | 2.14 ± 0.57 | 0.36 ± 0.11 | 0.35 ± 0.09 | 0.42 ± 0.15 | 0.62 ± 0.17 | 5.63 ± 1.44 |
|  | 15 | 45 | 4.23 ± 0.08 | 1.30 ± 0.27 | 0.28 ± 0.36 | 2.44 ± 0.34 | 0.31 ± 0.05 | 0.45 ± 0.29 | 0.37 ± 0.02 | 0.42 ± 0.22 | 5.58 ± 0.93 |
|  | 15 | 55 | 4.28 ± 0.05 | 1.29 ± 0.19 | 0.30 ± 0.34 | 2.04 ± 0.29 | 0.34 ± 0.03 | 0.25 ± 0.05 | 0.29 ± 0.06 | 0.58 ± 0.1 | 5.09 ± 0.87 |
|  | 15 | 65 | 4.28 ± 0.02 | 1.20 ± 0.57 | 0.00 ± 0.00 | 2.30 ± 0.24 | 0.40 ± 0.03 | 0.22 ± 0.04 | 0.15 ± 0.17 | 0.61 ± 0.04 | 4.86 ± 0.44 |
|  | 15 | 75 | 4.30 ± 0.03 | 1.39 ± 0.14 | 0.00 ± 0.01 | 2.29 ± 0.23 | 0.38 ± 0.02 | 0.22 ± 0.04 | 0.23 ± 0.03 | 0.56 ± 0.05 | 5.08 ± 0.32 |

Table S7: Organic acids and pH after pretreatment of unwashed and washed 37^o^C samples (ND = Not detected)

| Storage duration | Pretreatment retention time (min) | Nominal storage moisture (%) | pH | Acetic acid (% DM) | Lactic acid (% DM) | Isobutyric acid (% DM) | Formic acid (% DM) | Malic acid (% DM) | Pyruvic acid (% DM) | Tartaric acid (% DM) | Total acids (% DM) |
| --- | --- | --- | --- | --- | --- | --- | --- | --- | --- | --- | --- |
| Day 220 | 5 | 25 | 4.50 ± 0.05 | 0.71 ± 0.33 | 1.73 ± 0.28 | 1.22 ± 0.99 | 0.21 ± 0.14 | 0.15 ± 0.14 | 0.23 ± 0.01 | 0.85 ± 0.83 | 5.11 ± 1.05 |
|  | 5 | 35 | 4.45 ± 0.06 | 0.44 ± 0.02 | 2.27 ± 0.72 | 0.80 ± 0.17 | 0.12 ± 0.02 | 0.16 ± 0.01 | 0.20 ± 0.01 | 0.26 ± 0.02 | 4.24 ± 0.86 |
|  | 5 | 45 | 4.30 ± 0.09 | 0.65 ± 0.38 | 3.08 ± 0.15 | 1.60 ± 0.72 | 0.21 ± 0.10 | 1.79 ± 2.42 | 0.33 ± 0.20 | 0.16 ± 0.22 | 7.82 ± 3.74 |
|  | 5 | 55 | 4.28 ± 0.01 | 0.23 ± 0.32 | 3.80 ± 0.53 | 1.13 ± 0.28 | 0.14 ± 0.04 | 1.18 ± 1.48 | 0.30 ± 0.17 | 0.09 ± 0.13 | 6.88 ± 0.35 |
|  | 5 | 65 | 4.16 ± 0.01 | 0.40 ± 0.04 | 2.89 ± 0.06 | 0.00 ± 0.00 | 0.11 ± 0.01 | 0.04 ± 0.00 | 0.12 ± 0.00 | 0.01 ± 0.01 | 3.56 ± 0.00 |
|  | 5 | 75 | 4.69 ± 0.03 | 0.53 ± 0.11 | 3.45 ± 0.48 | 0.00 ± 0.01 | 0.15 ± 0.04 | 0.09 ± 0.01 | 0.14 ± 0.05 | 0.04 ± 0.03 | 4.40 ± 0.72 |
|  | 10 | 25 | 4.34 ± 0.03 | 0.40 ± 0.57 | 1.91 ± 0.26 | 1.29 ± 0.01 | 0.21 ± 0.00 | 0.14 ± 0.03 | 0.22 ± 0.06 | 0.27 ± 0.38 | 4.46 ± 0.34 |
|  | 10 | 35 | 4.30 ± 0.01 | 0.74 ± 0.16 | 2.83 ± 0.59 | 1.65 ± 0.22 | 0.25 ± 0.05 | 0.19 ± 0.04 | 0.22 ± 0.03 | 0.27 ± 0.09 | 6.15 ± 0.94 |
|  | 10 | 45 | 4.18 ± 0.00 | 0.53 ± 0.74 | 3.52 ± 0.61 | 1.89 ± 0.48 | 0.27 ± 0.09 | 0.39 ± 0.12 | 0.24 ± 0.01 | 0.20 ± 0.01 | 7.04 ± 1.82 |
|  | 10 | 55 | 4.15 ± 0.04 | 0.37 ± 0.52 | 3.77 ± 0.14 | 1.42 ± 0.59 | 0.18 ± 0.09 | 1.33 ± 1.49 | 0.35 ± 0.28 | 0.00 ± 0.00 | 7.42 ± 3.12 |
|  | 10 | 65 | 4.03 ± 0.02 | 0.55 ± 0.08 | 2.96 ± 0.09 | 1.46 ± 0.07 | 0.10 ± 0.14 | 1.07 ± 1.18 | 0.26 ± 0.21 | 0.01 ± 0.01 | 6.41 ± 1.78 |
|  | 10 | 75 | 4.51 ± 0.03 | 0.78 ± 0.26 | 3.61 ± 0.86 | 2.15 ± 0.64 | 0.29 ± 0.16 | 0.62 ± 0.40 | 0.15 ± 0.03 | 0.01 ± 0.02 | 7.62 ± 1.56 |
|  | 15 | 25 | 4.23 ± 0.08 | 0.87 ± 0.06 | 1.63 ± 0.35 | 1.88 ± 0.39 | 0.28 ± 0.05 | 0.21 ± 0.02 | 0.23 ± 0.05 | 0.22 ± 0.21 | 5.32 ± 0.72 |
|  | 15 | 35 | 4.09 ± 0.01 | 0.40 ± 0.56 | 2.16 ± 0.50 | 1.31 ± 1.08 | 0.21 ± 0.13 | 0.29 ± 0.34 | 0.19 ± 0.02 | 0.13 ± 0.12 | 4.67 ± 2.75 |
|  | 15 | 45 | 3.96 ± 0.00 | 0.66 ± 0.10 | 2.72 ± 0.03 | 1.96 ± 0.07 | 0.24 ± 0.00 | 0.68 ± 0.53 | 0.17 ± 0.01 | 0.30 ± 0.19 | 6.72 ± 0.92 |
|  | 15 | 55 | 4.01 ± 0.01 | 0.43 ± 0.61 | 3.60 ± 0.20 | 1.66 ± 1.01 | 0.20 ± 0.13 | 0.75 ± 0.42 | 0.18 ± 0.05 | 0.10 ± 0.14 | 6.93 ± 1.73 |
|  | 15 | 65 | 3.90 ± 0.03 | 0.59 ± 0.21 | 3.05 ± 0.51 | 0.86 ± 1.22 | 0.22 ± 0.12 | 0.29 ± 0.17 | 0.16 ± 0.01 | 0.01 ± 0.01 | 5.18 ± 1.19 |
|  | 15 | 75 | 4.37 ± 0.08 | 0.87 ± 0.23 | 3.50 ± 0.28 | 1.32 ± 1.87 | 0.31 ± 0.02 | 0.69 ± 0.63 | 0.18 ± 0.03 | 0.00 ± 0.00 | 6.87 ± 2.49 |
|  | WASHED |  |  |  |  |  |  |  |  |  |  |
| Day 220 | 15 | 25 | 4.00 ± 0.01 | 0.83 ± 0.08 | 0.29 ± 0.01 | ND | 0.38 ± 0.02 | 1.78 ± 2.10 | 0.23 ± 0.15 | ND | 3.51 ± 2.14 |
|  | 15 | 35 | 3.96 ± 0.01 | 0.89 ± 0.00 | 0.37 ± 0.09 | ND | 0.34 ± 0.01 | 1.93 ± 2.23 | 0.22 ± 0.17 | ND | 3.74 ± 2.51 |
|  | 15 | 45 | 3.95 ± 0.06 | 0.87 ± 0.01 | 0.38 ± 0.09 | ND | 0.38 ± 0.04 | 2.42 ± 2.83 | 0.08 ± 0.04 | ND | 4.13 ± 2.93 |
|  | 15 | 55 | 3.86 ± 0.01 | 0.91 ± 0.02 | 0.32 ± 0.01 | ND | 0.31 ± 0.00 | 2.90 ± 2.23 | 0.23 ± 0.11 | ND | 4.67 ± 2.31 |
|  | 15 | 65 | 3.82 ± 0.02 | 0.79 ± 0.04 | 0.41 ± 0.14 | ND | 0.28 ± 0.03 | 3.26 ± 0.10 | 0.30 ± 0.02 | ND | 5.04 ± 0.02 |
|  | 15 | 75 | 3.96 ± 0.00 | 0.93 ± 0.06 | 0.17 ± 0.07 | ND | 0.34 ± 0.03 | 0.37 ± 0.01 | 0.09 ± 0.00 | ND | 2 .00 ±0.10 |

Figure S3: Change in amounts of organic acids from unwashed wet stored samples and total amount of acids in pretreatment extracts for three pretreatment times (5, 10 and 15 minutes) and six moisture contents (25% to 75% moisture, wet basis).

Changes in organic acid profile are percentage point difference represented by ‘acids present in pretreatment extract (%) - storage acids (%)’, using mean values, all on a dry matter basis. Negative values indicate the amount of acid after pretreatment was less than the amount in the wet storage sample prior to pretreatment. Total acids (triangles with error bars) are also read from the same axis and include other minor acids present in the pretreatment extract. Error bars are standard deviation of means.

Table S8: Concentration of potential inhibitors in pretreatment extract of unwashed samples stored at 23^o^C†

† Includes data from replicates to be fermented with and without the pretreatment extract

Table S9: Concentration of potential inhibitors in pretreatment extract of unwashed and washed samples stored at 37^o^C

Table S10: Summary stats on concentration of potential inhibitors in unwashed samples stored at 23^o^C, averaged across moisture

|  | Concentration of potential inhibitors | | | | |
| --- | --- | --- | --- | --- | --- |
|  | Storage | Mean | StDev | Minimum | Maximum |
| Lactic acid, g/L | Ensiled | 3.68 | 1.04 | 1.00 | 5.25 |
|  | Unensiled | 0.18 | 0.39 | 0.00 | 1.47 |
| Acetic acid, g/L | Ensiled | 0.81 | 0.42 | 0.00 | 1.69 |
|  | Unensiled | 1.31 | 0.41 | 0.27 | 2.62 |
| HMF, g/L | Ensiled | 0.05 | 0.02 | 0.01 | 0.10 |
|  | Unensiled | 0.04 | 0.02 | 0.00 | 0.11 |
| Furfural, g/L | Ensiled | 0.59 | 0.33 | 0.10 | 1.26 |
|  | Unensiled | 0.33 | 0.22 | 0.00 | 0.76 |

Table S11: Difference in pH units before and after pretreatment. †

† Note that each unit difference in pH represents a tenfold change (increase or decrease) in acidity

Table S12: Ethanol yield and inhibitor concentrations† in pretreated unwashed stover fermented with pretreatment extract

† Concentrations are grams per liter of fermentation broth. Since ethanol yields are calculated as percentage of the theoretical yield possible from the glucose present in the feedstock at the time of fermentation (Equation 1), these yields can be compared with fermentation of samples without the liquid extract. The amount of glucose for samples fermented with extract includes glucose in the pretreatment solid fraction and in the liquid extract.

Table S13: Ethanol yield and inhibitor concentrations† in pretreated unwashed and washed stover stored at 37^o^C, fermented with pretreatment extract

† Concentrations are grams per liter of fermentation broth

*Data missing

Table S14: Ethanol yield from pretreated unwashed and washed stover fermented without pretreatment extract and potential ethanol lost with extract†

† Ethanol yield for samples fermented without extract was calculated after subtracting the glucan removed into the liquid extract during pretreatment. That is, the amount of glucose used in calculating expected or theoretical yield is the amount in the pretreatment solid fraction only.

The potential ethanol calculated from this extract provides an estimate of the increase in theoretical ethanol yield possible in a commercial facility where the extract would likely never be separated from the solids during or after pretreatment. See Equation 2 used in calculating potential ethanol as percentage of broth total, which is theoretical ethanol yield if pretreatment solids and extracts were used. *Not part of experimental design

Equation 1:

$$Ethanol yield (\% of theoretical)=\frac{{EtOH}_{actual}}{\frac{\left( G_{extract}+G_{solid} \right)}{0.9}\times0.51} \times100$$

Where:

EtOH_actual_ = actual amount of ethanol in fermentation broth as measured using the YSI 2700 SELECT (g)

G_extract_ = Amount of glucan in pretreatment extract (g)

(When samples are fermented without extract, G_extract_ = 0)

G_solid_ = Amount of glucan in pretreated dry matter that is fermented (g)

= [(% glucan before pretreatment ×dry mass of feedstock before pretreatment (g)) - Amount of glucan in pretreatment extract (g)]

0.9 = (molar mass of glucan/molar mass of glucose) is to convert glucan to glucose

0.51 = conversion factor of 1 gram of glucose to ethanol from stoichiometric

(Used as basis for theoretical yield)

$$C_{6}H_{12}O_{6}\to{2C}_{2}H_{5}OH+2CO_{2}$$

$$Glucose \left( 180g \right)\to Ethanol\left( 2\times46g \right)+Carbon-dioxide (2\times44g)$$

Equation 2:

$$\% potential ethanol from extract=\frac{\frac{G_{extract}}{0.9}\times0.51}{\frac{\left( G_{extract}+G_{solid} \right)}{0.9}\times0.51} \times100$$

Where: G_extract_ and G_solid_ are defined as in Equation 1

Table S15: Correlation between ethanol yield (% of theoretical) and potential inhibitors generated during pretreatment of unwashed unensiled stover†

|  | Ethanol (%) | Furfural (g/L) | HMF (g/L) | Acetic (g/L) | Lactic (g/L) |
| --- | --- | --- | --- | --- | --- |
| Furfural (g/L) | 0.852 |  |  |  |  |
|  | 0.000 |  |  |  |  |
|  |  |  |  |  |  |
| HMF (g/L) | 0.657 | 0.871 |  |  |  |
|  | 0.000 | 0.000 |  |  |  |
|  |  |  |  |  |  |
| Acetic (g/L) | 0.659 | 0.726 | 0.432 |  |  |
|  | 0.000 | 0.000 | 0.009 |  |  |
|  |  |  |  |  |  |
| Lactic (g/L) | 0.163 | 0.293 | 0.511 | -0.008 |  |
|  | 0.341 | 0.082 | 0.001 | 0.965 |  |
|  |  |  |  |  |  |
| Isobutyric (g/L) | 0.826 | 0.916 | 0.680 | 0.824 | 0.164 |
|  | 0.000 | 0.000 | 0.000 | 0.000 | 0.340 |
|  |  |  |  |  |  |
|  | Cell content: Pearson correlation (top) | | | |  |
|  |  | P-Value (beneath) | |  |  |
|  |  |  |  |  |  |

† Concentrations of potential inhibitors are in grams per liter of fermentation broth

Table S16: Correlation between ethanol yield (% of theoretical) and potential inhibitors generated during pretreatment of unwashed ensiled stover stored for 220 days†

|  | Ethanol (%) | Furfural (g/L) | HMF (g/L) | Acetic (g/L) | Lactic (g/L) |
| --- | --- | --- | --- | --- | --- |
| Furfural (g/L) | 0.826 |  |  |  |  |
|  | 0.000 |  |  |  |  |
|  |  |  |  |  |  |
| HMF (g/L) | 0.750 | 0.904 |  |  |  |
|  | 0.000 | 0.000 |  |  |  |
|  |  |  |  |  |  |
| Acetic (g/L) | 0.247 | 0.343 | 0.339 |  |  |
|  | 0.146 | 0.040 | 0.043 |  |  |
|  |  |  |  |  |  |
| Lactic (g/L) | 0.330 | 0.315 | 0.195 | -0.115 |  |
|  | 0.049 | 0.062 | 0.255 | 0.505 |  |
|  |  |  |  |  |  |
| Isobutyric (g/L) | 0.399 | 0.562 | 0.627 | 0.208 | 0.271 |
|  | 0.016 | 0.000 | 0.000 | 0.223 | 0.110 |
|  |  |  |  |  |  |
|  | Cell content: Pearson correlation (top) | | | |  |
|  |  | P-Value (beneath) | |  |  |
|  |  |  |  |  |  |

† Concentrations of potential inhibitors are in grams per liter of fermentation broth
